# Effective surveillance systems for vector-borne diseases in urban settings and translation of the data into action: a scoping review

**DOI:** 10.1186/s40249-018-0473-9

**Published:** 2018-09-03

**Authors:** Florence Fournet, Frédéric Jourdain, Emmanuel Bonnet, Stéphanie Degroote, Valéry Ridde

**Affiliations:** 1 Infectious Diseases and Vectors Ecology, Genetics, Evolution and Control (MIVEGEC), French National Research Institute for Sustainable Development, 911 Avenue Agropolis, BP 64501, 34394 Montpellier Cedex 5, France; 2 Résiliences, French National Research Institute for Sustainable Development, 32 Avenue Henri Varagnat, 93140 Bondy, France; 3 University of Montreal, Public Health Research Institute, 7101 avenue du Parc, Montréal, Québec Canada; 4 Population and Development Center (CEPED), French National Research Institute for Sustainable Development, Université Paris Sorbonne, 45, rue des Saints Pères, 75006 Paris, France

**Keywords:** Vector-borne diseases, Surveillance systems, Urban health, Scoping review

## Abstract

**Background:**

Vector-borne diseases (VBDs) continue to represent a global threat, with “old” diseases like malaria, and “emergent” or “re-emergent” ones like Zika, because of an increase in international trade, demographic growth, and rapid urbanization. In this era of globalization, surveillance is a key element in controlling VBDs in urban settings, but surveillance alone cannot solve the problem. A review of experiences is of interest to examine other solution elements. The objectives were to assess the different means of VBD surveillance in urban environments, to evaluate their potential for supporting public health actions, and to describe the tools used for public health actions, the constraints they face, and the research and health action gaps to be filled.

**Main body:**

For this scoping review we searched peer-reviewed articles and grey literature published between 2000 and 2016. Various tools were used for data coding and extraction. A quality assessment was done for each study reviewed, and descriptive characteristics and data on implementation process and transferability were analyzed in all studies.

After screening 414 full-text articles, we retained a total of 79 articles for review. The main targets of the articles were arboviral diseases (65.8%) and malaria (16.5%). The positive aspects of many studies fit within the framework of integrated vector management. Public awareness is considered a key to successful vector control programs. Advocacy and legislation can reinforce both empowerment and capacity building. These can be achieved by collaboration within the health sector and with other sectors. Research is needed to develop well designed studies and new tools for surveillance and control.

**Conclusions:**

The need for surveillance systems in urban settings in both developing and developed countries was highlighted. Countries face the same challenges relating to human, financial, and structural resources. These findings also constitute a wake-up call for governments, academia, funders, and World Health Organization to strengthen control programs and enhance VBD research in urban environments.

**Electronic supplementary material:**

The online version of this article (10.1186/s40249-018-0473-9) contains supplementary material, which is available to authorized users.

## Multilingual abstract

Please see Additional file 1 for translations of the abstract into the five official working languages of the United Nations.

## Background

Vector-borne diseases (VBDs) account for over 17% of all infectious diseases, resulting every year in more than one billion cases and over one million deaths [1]. The burden of these diseases is linked to the challenges of prevention and control, particularly because there is no vaccine for most of them. Moreover, distribution of these diseases is determined by a complex dynamic of environmental and social factors. Globalization of travel and trade, unplanned urbanization, migration, and environmental challenges such as climate change have had a significant impact on disease transmission in recent years, with major outbreaks of dengue, chikungunya, and Zika virus.

Though some VBDs tend to be perceived as rural diseases, there is evidence pointing to the transmission in urban settings of malaria [2], Chagas disease [3], and sleeping sickness [4]. Other diseases, and especially *Aedes*-borne diseases (dengue, chikungunya, Zika), are mainly an urban challenge because of unplanned and extensive urbanization, invasion by different vectors (*Ae. aegypti*, and *Ae. albopictus*), and the globalization of commerce and travel [5, 6]. New opportunities for VBDs to flourish and spread are created in the cities of the developing world, compromising the well-being of populations [7].

In high-income countries, the main issue is to prevent the introduction of diseases that may cause an epidemic or re-emergence. As an example, the recent Zika outbreak highlighted the need for an early warning system and preparedness [8], while the issue of the resurgence of malaria is recurrent in Europe [9]. In low- and middle-income countries, control and early detection of outbreaks is needed. Dealing with the dangers of VBDs in developed and developing countries requires strong surveillance systems and effective interventions. An effective surveillance system should be able to collect and analyze data to produce information and disseminate it to those who can promote public health policies and relevant prevention and control strategies. By strengthening the World Health Organization (WHO)’s capacity to assess the public health value of new vector control tools and technologies and develop appropriate technical recommendations, the newly established Vector Control Advisory Group supports national and global efforts to control and eliminate VBDs worldwide.^1^

We undertook a scoping review to examine the different means of VBD surveillance in urban environments, and to evaluate their potential for supporting public health actions. The tools used for public health actions, the constraints they face were highlighted for identifying the research and the health action gaps to be filled.

## Methods

### Use of the eDelphi process to select scoping review topics

Using an eDelphi survey, we invited a panel of 109 international experts (43% researchers; 52% public health decision-makers; 5% private sector experts) to identify the six topics of highest priority [10]. The survey involved three rounds: 1) participants suggested topics; 2) the more than 80 topics suggested were then rated from “1 – eliminate” to “5 – top priority”; and 3) the 20 subjects rated 4 or 5 by more than 65% of the participants were rated a second time. At the end of the third round, the present topic obtained the mean rating of 4.00 ± 1.02 and was ranked 5th (rated 4 or 5 by 71.43% of the participants).

### Search strategy

We conducted a systematic search in MEDLINE, Embase, Global Health, and Web of Science in August–September 2016 to identify published studies. The search strategy was validated by a public health librarian and consisted of combining the following concepts using associated keywords and descriptors: vector-borne diseases, urban setting, surveillance system, and public health actions (see full list in Additional file 2). Additional articles were identified by manually screening the references of papers that met our inclusion criteria.

### Study selection

Three investigators (EB, FJ, and FF) independently screened all titles and abstracts using defined inclusion criteria: 1) was published between 2000 and 2016; 2) concerned any vector or VBD listed by WHO in 2016^2^; 3) was written in English, French, or Spanish; 4) had an available abstract; 5) dealt with any aspect of VBD surveillance (vector, human, animal, or environmental surveillance); 6) described surveillance outcomes (i.e., implementation or possibility of implementation of public health actions); 7) was related to urban populations or implementation at the country level for VBDs with serious urban potential (i.e., dengue). Excluded were: secondary reports; editorial opinions; personal communications; studies that were purely descriptive with no quantitative or qualitative analysis; studies with only one outcome of interest (surveillance OR public health action OR urban setting); studies without the notion of effectiveness or focusing on a limited monitoring period (generally the case with cross-sectional and case-control studies); studies aimed at testing a new vector control tool (insecticide, repellent or new trap); and studies about surveillance in rural areas. The same investigators (EB, FJ, and FF) reviewed full-text articles for inclusion, with disagreement settled by consensus.

### Studies’ characteristics, quality assessment, and data extraction

Descriptive characteristics, quality assessment, and data from articles meeting the inclusion criteria were extracted into a standardized template using a Microsoft Excel 2013 (Microsoft Corporation, Washington, Etats Unis) spreadsheet that was validated by two contributors with agreement on over 85% of data extracted from the same three studies.

First, the quality of the studies was assessed with the Mixed Methods Appraisal Tool (MMAT) [11]. The MMAT has been designed for the appraisal stage of complex systematic literature reviews. The first criteria could be applied whatever the study (clear objectives and correctly addressed question), though the following depend on the study type: qualitative, quantitative and mixed methods studies. They aimed to cross the data sources, the method of the data collection or the population recruitment. Studies were ranked based on the extent to which they satisfied specific criteria; they were labelled *yes*, *no*, or *can’t tell* or *not applicable*, depending on whether they clearly met the criteria, or whether it was not possible to determine from the reporting whether they met them, or if the criteria were not relevant to the purpose of the study (see Additional file 3). For the evaluation, scores of 4, 3, 2, and 1 were applied to the answers *yes*, *no*, *can’t tell* and *not applicable*, respectively. Completeness of intervention description was assessed using the Template for Intervention Description and Replication (TIDieR) checklist [12]. The TIDieR checklist was used to document the rationale, materials, procedures (how, by whom, when, and where the intervention took place), modifications, and fidelity of the intervention (see Additional file 3). To synthesize the findings from the included studies, we used Analysis of Transferability and Support to Adaptation of Health Promotion Interventions (ASTAIRE) [13]. ASTAIRE tool examines 23 criteria which are divided into four broad categories of elements that describe the population, the environmental factors that can influence the effects of the intervention, the implementation of the intervention according to the policies and the partnership, the accompaniment to the transfer of the intervention to adapt context (see Additional file 3).

## Results

### Description of included studies

**Fig. 1:**
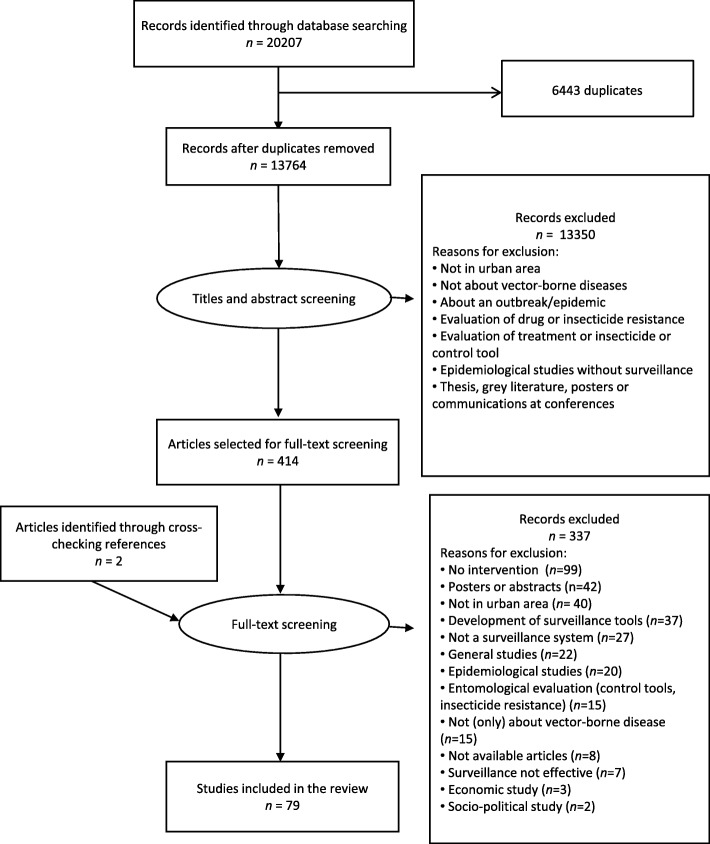
Prisma flow chart of article search and selection

Our search strategy yielded 20 207 documents. Of those, 6443 duplicates were removed, leaving 13 764 articles to screen. Title and abstract screening led to the selection of 414 documents, of which 77 met our inclusion criteria after full-text screening (see Fig. 1). Two documents were added after cross-checking references. All documents included were peer-reviewed articles.

**Fig. 2:**
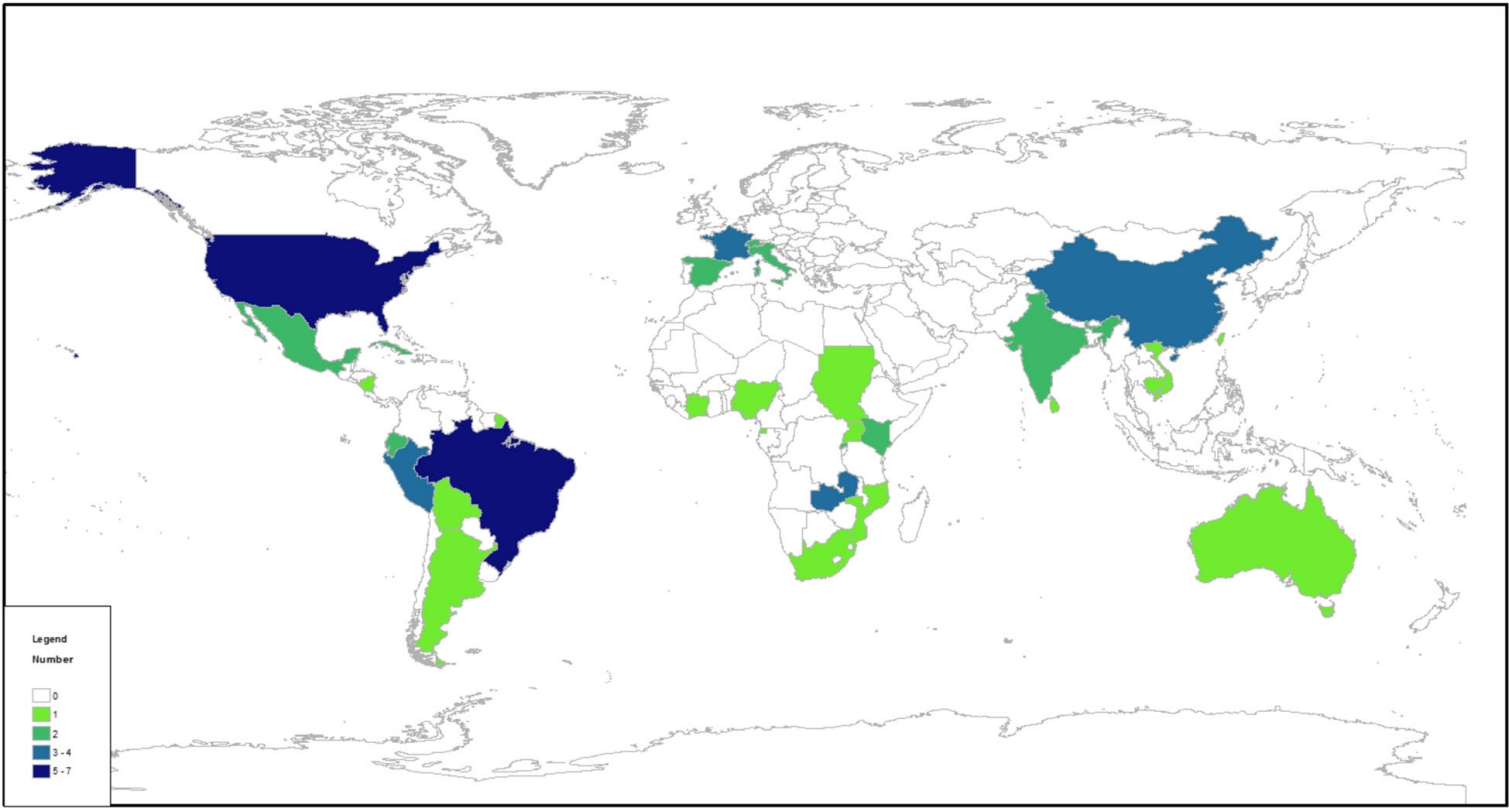
Distribution of the studies by countries

Studies were carried out in Latin America (25.3%; *n =* 20), Africa (19.0%; *n =* 15), Asia (19.0%; *n =* 15), the USA (13.9%; *n =* 11), Europe (12.7%; *n =* 10), and Oceania (8.9%; *n =* 7) (se Fig. 2). One article concerned two continents [14]. Diseases included malaria (16.5%; *n =* 13), dengue fever (35.4%; *n =* 28), chikungunya fever (3.8%; *n* = 3), yellow fever (2.5%; *n =* 2), Zika virus (2.5%; *n =* 2), West Nile fever (11.4%; *n =* 9), Chagas disease (8.9%; *n =* 7), leishmaniasis (2.5%; *n =* 2), sleeping sickness (1.3%; *n =* 1), filariasis (2.5%; *n =* 2), Lyme disease (1.3%; *n =* 1), and schistosomiasis (1.3%; *n =* 1). The eight (10.1%) remaining articles focused on mosquitoes in general (*n =* 1) or *Aedes* (*n =* 7).

**Fig. 3:**
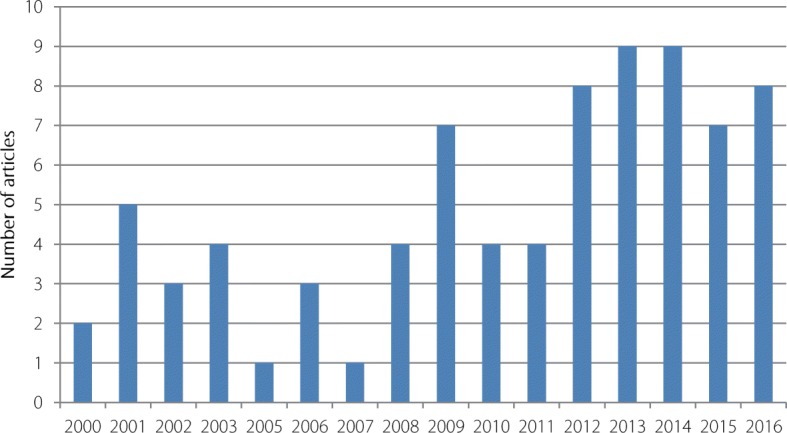
Evolution of scientific production by year

Scientific production has doubled since 2010, which corresponds to the emergence and re-emergence of arboviral diseases globally (see Fig. 3).

**Fig. 4:**
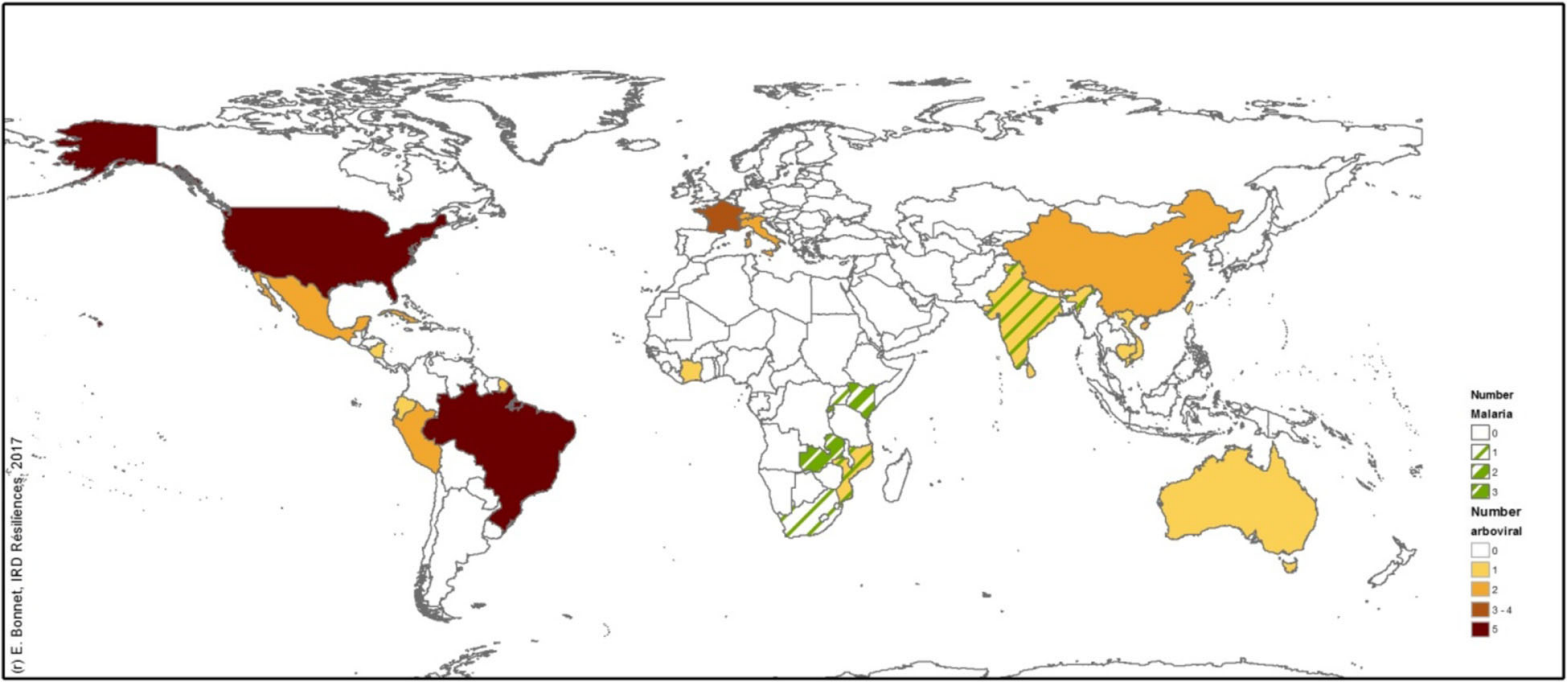
Distribution of studies on malaria and arboviral transmission by countries

Malaria was reported only in Africa (*n =* 12) and in India (*n =* 1) (see Fig. 4). Arboviral diseases strongly affected Asia and Latin America. High-income countries such as the USA and European countries were affected by specific diseases such as West Nile and Lyme diseases, as well as by *Aedes*-borne diseases (dengue, chikungunya, and Zika).

Characteristics of the 79 included studies are detailed in Additional file 4. The surveillance tool, its target and objectives, the type and target of the intervention implemented, an overview of the results, the lessons learned from the intervention, as well as the main limitations of the process are reported.

### Quality of studies included

**Fig. 5:**
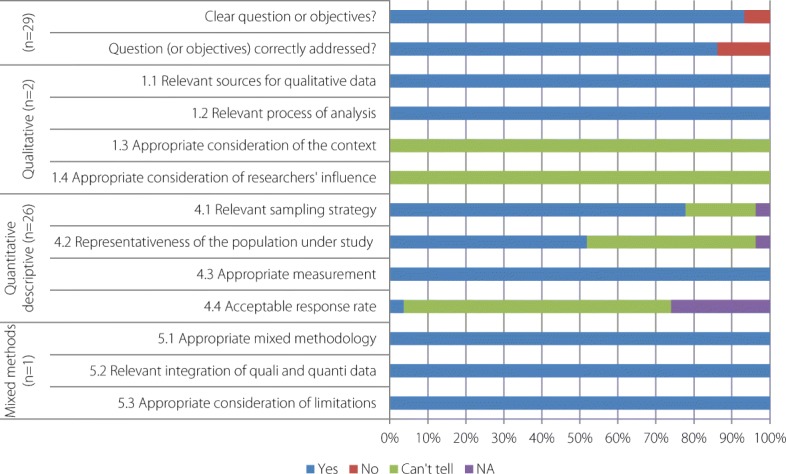
Quality assessment of the 30 studies evaluating through MMAT

Only 30 of the 79 articles (38%) were evaluated with the MMAT (see Additional file 3 and Fig. 5). All of the 49 articles (62%) that could not be evaluated with the MMAT were classified as “non research studies” since they did not use any analytical method. These articles could be classified as reviews (38.8%; *n =* 19), reports of cases or outbreaks (34.7%; *n* = 17), or epidemiological updates (26.5%; *n =* 13), but all were peer-reviewed.

The 30 studies evaluated by MMAT consisted of two qualitative studies, one mixed-methods study, and 27 quantitative descriptive studies. Evaluation of the overall quality of the studies with MMAT produced a mean score of 85.7% and a median of 87.5%.

### Types of interventions

**Fig. 6:**
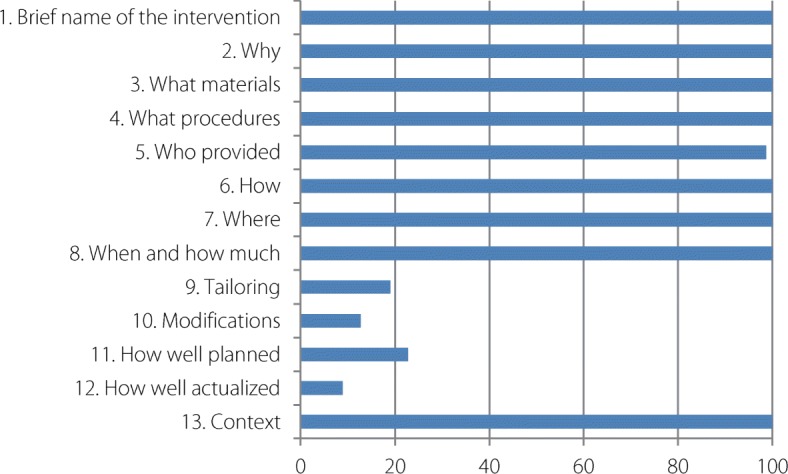
Percentage of studies reporting elements of description of the interventions according to the TIDieR tool

The TIDieR checklist was used to document the rationale, materials, procedures (how, by whom, when, and where the intervention took place), modifications, and fidelity of the intervention (see Additional file 3). Figure 6 illustrates to what extent the interventions were described in each study included.

Interventions were divided into those targeting disease transmission (for example, by distributing mosquito nets, administering artemisinin-based combination therapy [ACT] more widely in the case of malaria, or reducing vector sources) [15–18], those improving case identification [19], and those implementing warning systems to limit the spread of the disease, such as for the West Nile [20–22] or Zika virus [23]. In some cases, vector sensitivity to insecticides [24] or pathogen sensitivity to drugs [25] were surveyed, leading to adaptation of the interventions.

Interventions can also determine the riposte framework, which may involve improving the case definition, initiating mandatory reporting (arboviral diseases), or constraining the population to reduce the vector source (container protection, waste management, etc.) or even to participate financially in the intervention, as in Singapore [26].

### Implementation process and transferability

**Fig. 7:**
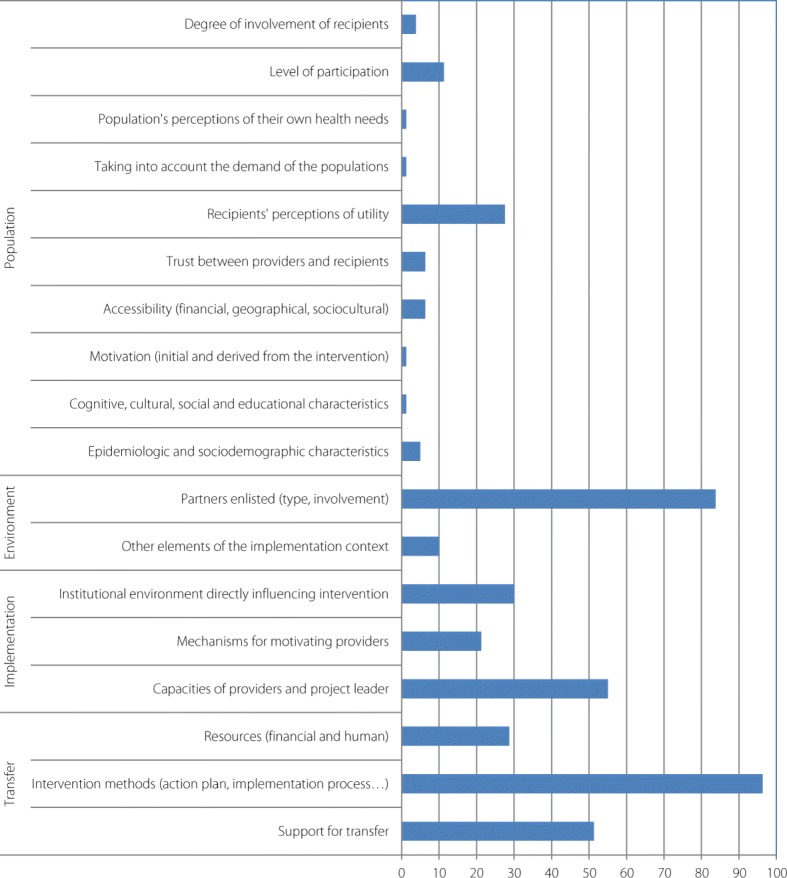
Percentage of studies (*n =* 79) reporting elements listed in the ASTAIRE tool

Figure 7 illustrates the availability of descriptions of the interventions’ contexts according to the ASTAIRE tool, which is useful information for transferability purposes.

### Evaluation of the surveillance and of intervention effectiveness

#### Different kinds of surveillance systems

Surveillance systems were either active (25.3%; *n =* 20), as in Ticino (Italy) [27], passive (46.8%; *n =* 37), as in Mutale municipality (South Africa) [28], or both (27.8%; *n =* 22), as in the Luba focus on Bioko Island (Equatorial Guinea) [29]. Surveillance targeted the vector (29.1%; *n =* 23) [27], the pathogen (10.1%; *n =* 8) [30], or the human population (cases, patients, travelers) (30.4%; *n =* 24) [23]. In cases involving active surveillance, vectors were surveyed in 55% (11/20) of studies, whereas in cases involving passive surveillance, they were surveyed in 24.3% (9/37) of the studies. In most of the studies, only one target (vector or human) was surveyed (69.6%; *n =* 55). In 18 studies, there were two targets, generally the vector and the human population (22.8%; *n =* 18). Only five studies surveyed vector, human, and/or animal populations and pathogens (6.3%; *n =* 5) [31–35]. Integrated surveillance systems taking into account all actors of the pathogen system were rarely implemented, or at least were rarely described in the reviewed articles.

Surveillance also led to better knowledge about vectors (spatiotemporal distribution, sensitivity to insecticides, existence of a secondary reservoir [36, 37]) or pathogens (identification of the dengue serotypes in circulation [38]).

The municipal scale was considered in 39.2% of the studies (*n =* 31). The other studies were conducted at the region level (17.7%; *n =* 14), the country level (41.8%; *n =* 33), and even the European level, in the case of Kampen et al. [39] (1.3%; *n =* 1).

#### Effectiveness of the intervention

More than 50% (*n =* 41) of the 79 studies reported that the intervention was effective. The other studies did not aim to measure intervention effectiveness. In 75.9% (*n =* 60) of the studies, the intervention was based only on vector control (46.8%; *n =* 37) or on other measures focused on the human population, the pathogen, or the environment.

Various outcomes were used to measure intervention effectiveness: epidemiological variables, such as disease prevalence or incidence (53.1%; *n =* 42); entomological indices, such as the container index, Breteau index, or rate of eliminated containers (55.7%; *n =* 44); and vaccination or mosquito net coverage (11.4%; *n =* 9). Other outcomes included the production of guidelines [31, 40] or the development of policy decisions [20–22, 41, 42].

Generally speaking, the importance of institutional support and partner mobilization, both key elements of an effective integrated vector management strategy were highlighted. Some positive outcomes concerned the human population, who adopted better behaviours such as proper covering of water supplies [27, 32], elimination of *Aedes* breeding sites [37, 43], or increase in hospital use [44]. In Brazil, a hand-made mosquito ovitrap brought added value to the intervention [45]. People were able to learn about mosquito biology, contribute to the intervention by preventing mosquito proliferation, and provide clear images of their environment with minimal financial investment. Presenting the health message in different languages also appeared to favour sensitization [26]. At the health team level, improvements were also observed. In some cases, thanks to a participatory process, data collection tools were improved [32] and new tools, such as Global Positioning System or smartphones, were used that enhanced the process by reducing the time lag between data collection and dissemination [39, 46]. Lee et al. [23] showed that experience with Ebola outbreaks and the West Nile virus in the USA was later useful for developing and implementing plans for managing Zika virus.

In some studies, the authors reported that entomological indices did not decrease; this result was attributed to persistent breeding sites that were not surveyed, or to residual transmission [20–22, 40, 47–49]. Some variations in intervention impacts were reported in the surveyed areas as being due to environmental heterogeneity [50], unequal mobilization [51], or variable intervention coverage [52]. Limitations in impact were also attributed to an absence of intervention in adjacent area [28, 53]. One study showed a negative impact of the intervention, which induced vector resistance to the insecticide used [37]. In another study, the intervention highlighted that the pathogen was resistant to the treatment, leading to the promotion of new treatment guidelines [25].

### Challenges faced

Several challenges were identified that were mainly linked to the operational chain, which needs to be precisely laid out, with guidelines for case definition, diagnosis, and data collection [51], and known to all actors [54]. The lack of human, financial, and infrastructural capacity was another challenge [55–58], as was the low level of involvement of decision-makers, which explained the paucity of political will [24]. This poor political will often caused a time lag between data collection and dissemination [40]. Lastly, lack of community involvement appeared as a key challenge to ensuring both the effectiveness and especially the sustainability of the control [51, 59, 60]. Taleo et al. [61], pointed out that “dengue is often a problem generated by communities themselves and, as such, the solution is in their hands”, while another study asserted that “the success of any prevention program depends on either convincing individuals to change their behaviour or changing the environment to remove factors that place individuals at risk of disease” [49]. Moreover, community mobilization cannot be achieved if the political authorities of the countries are not also involved in the activities. The challenge is therefore to reinforce people’s involvement through a better awareness of the disease, because “a low risk perception in the community underestimates the high danger potential of vector-borne diseases, which also may impact the effectiveness of public health interventions” [24].

### Lessons learned and recommendations

Recommendations included timely release of surveillance results to facilitate prompt remedial actions for vector control [62], health education to sustain public participation in vector prevention and control [60], scaling up the use of protective measures such as long-lasting impregnated nets [15], improving detection of cases [55, 63], implementing control tools that are adapted to the local context (including perceptions of field workers and communities) [14], and considering the role played by animals [36].

Interventions need to be sustainable, and consideration should be given to using new and cost-effective technologies such as geographic information systems (GIS) and mobile health or hand-held systems to improve field reporting [40, 45, 60, 64]. Only 26 studies mentioned the use of GIS (32.9%). Three studies highlighted the potential benefits of mobile phone use. Kampen et al. [39] achieved good mobilization of the population, referred to as citizen science, with the development of mobile phone apps such as Tigatrapp© in Spain or Imoustique© in France. Mobile phones were also used by Larsen et al. [65] to accelerate the reporting of malaria data in Zambia [52] and Brazil [64]. This reduced the time lag between data collection and their translation into operational actions, which is generally identified as an obstacle for good surveillance systems.

A multi-disease control approach could also benefit from improved communications, particularly in surveillance targeting several vectors [24, 57, 61, 66]. Likewise, more key informants should be involved, as well as ‘alternative’ medical clinics and traditional healers [61].

## Discussion

The review aimed to highlight the research and the health action gaps to be filled to better control vector-borne diseases in urban settings. The weak representation of malaria in the reviewed studies suggests that, even though this disease represents a threat in urban areas, it remains poorly tackled in these settings.

The positive aspects of many of the studies fit within the framework of integrated vector management (IVM), which is a rational decision-making process for the optimal use of resources for vector control [67, 68]. Promoting this framework should help to improve the effectiveness and sustainability of public health actions; the framework is well illustrated by certain key experiences encountered in this review, which are highlighted in the following sections.

### Advocacy, social mobilization, and legislation

Awareness among the different stakeholders is crucial. Public awareness improves people’s understanding of risks and of prevention strategies [23, 69] and is considered a key to success in dengue prevention programs [60, 70]. Communities’ capacity to participate effectively in the control of arbovirus vectors is well documented and is based mainly on behaviour modification and on mobilizing the community in controlling breeding sites [24, 32, 43, 60–62, 71–73]. Community-based larval control is also an approach that might be useful in towns and cities to control malaria [16].

High level-advocacy is needed to obtain state support for the program implementation phase [60], especially in emergency situations requiring high governmental investment [56]. The mobilization of all actors must be sustained by continuous dissemination of information to health professionals to promote good diagnostic and case management practices [57, 69]. Effective social mobilization requires a dedicated strategy that identifies main targets (schoolchildren, property managers, construction sites, local organizations, etc.) and key messages [69, 74]. Inadequate community involvement was found to be the main obstacle to the effectiveness of control programs to eliminate mosquitoes or bedbugs in certain cases where non-participating households were the main reservoirs for residual infestation [25, 43, 51]. Public awareness can usually be maintained by regular visits from health inspectors [43]. New technologies can be used to respond to various issues, but practices need to change. Here again, awareness is a prerequisite to overcoming resistance to change [46]. The perceptions and opinions of field staff also must be taken into account, upstream of program definition, to ensure operationality and acceptance [14].

Regulatory aspects are important, as control programs are part of an organizational and technical framework that needs to be framed by legislation [24]. Nevertheless, to go further, a political commitment is also needed and would be strengthened by legislation. Yoshikawa [26] describes, for example, the development of a legal framework to control *Aedes* vectors in Singapore. Legislative measures have been also implemented to limit the presence of breeding sites of malaria vectors in buildings and during construction [18].

### Collaboration within the health sector and with other sectors

Collaboration within the health sector is particularly relevant in cases of complex integrated surveillance, such as surveillance of the West Nile virus [41]. Actions to improve coordination among different health actors should be supported [75], especially by ensuring that actors involved in surveillance are connected with those conducting interventions [24]. Collaboration with other sectors, such as infrastructure construction, urban planning and management, and water and sanitation, fosters intersectoral management of vector-borne risk [18, 76]. Incorporating the private sector remains a challenge in the field of surveillance or control [66, 72]. This is particularly relevant in urban environments, where the dialogue between private construction companies and public authorities needs to be strengthened [49].

A critical strategy to encourage such collaboration would be the formalization and implementation of dedicated cross-sectoral coordination structures [18]. Collaboration between research and public health must be fostered to improve effectiveness and evaluation of surveillance and control programs, while taking account scale issues and operational constraints.

### Capacity building

The development of essential human resources through training is emphasized in every sector, from surveillance to disease control, to improve rapid detection and response to health events [18, 41, 49, 54, 77–79]. Capacity building also concerns infrastructure and equipment [18, 20], as well as technologies such as GIS [24, 34]. It is worth noting that capacities implemented in a specific context are an investment that can prove very useful in an emergency situation. For example, the Zika response in New York City relied upon emergency capacities first developed in 1999 during the West Nile virus outbreak [23]. In most cases, such capacity building is most efficient when developed at not only the national but also the local level [30, 32].

### Evidence-based decision-making

Surveillance data are the pillar of evidence-based intervention and need to be integrated and available in a timely manner [14, 24, 45, 46, 66, 76, 80]. To produce strategies and interventions that are appropriate, actors need to know the local vector ecology [81] and the epidemiological systems in their entirety, including the zoonotic cycle [31, 36, 53, 77], as well as the extent of potential secondary vectors, especially in a context of elimination [76, 82].

Most often, situations evolve in response to stimuli, such as insecticide resistance [37], introduction of an invasive vector [58, 83], or unexpected route of transmission [23]. In this context, much knowledge is needed, and strategies must be based on scientific evidence to be efficient and cost-effective [66].

### Need for innovative interventions and research

New technologies improve mapping and reporting [15, 66], but sustainable surveillance systems must be maintained and reinforced in terms of sensitivity and geographic coverage to detect weak points in control, to rationalize resources, or to contend with new challenges such as identifying the main locations of importation of cases [28, 84, 85], which may pose ethical and legal concerns [86]. Such approaches facilitate the integration of data from different surveillance system as well as the timely, efficient, and cost-effective deployment of focused interventions [15, 38, 45, 46, 64, 66, 69, 87–89]. These tools help to overcome difficulties that are frequently encountered in developing countries, such as poor urban planning and unregulated urban expansion [46], and can enable dissemination of surveillance results to the public for sensitization and mobilization [60, 59]. The use of GIS may also facilitate the development of spatial analysis and risk models, which enable the development of early warning systems [34, 45, 64, 72, 88].

The need for new control tools to remedy certain difficulties linked to drug resistance and insecticide resistance opens up new research purposes, such as vaccines and innovative vector control approach based on genetically modified mosquitoes [90]. Finally, as stated by WHO [91], innovation is essential in the field of vector control to address numerous challenges, such as insecticide resistance and the development of environmentally-friendly and vector-specific control methods [50, 92].

### Limitations of the study

We extracted more than 20 000 articles based on our search strategy, but even though we performed a double screening, some relevant articles may have escaped. The complete data extraction grid used for this review is available in Additional file 3.

Some studies were considered non-research studies, and could not be evaluated by the MMAT. In fact, the quality of these studies was not to question, but rather to improve this tool to expand its use. The design of our review, which targeted interventions based on surveillance systems and not just interventions in themselves, also complicated the identification of studies. Indeed, it was difficult to identify articles or documents dealing with surveillance systems and with public health actions actually implemented on the basis of surveillance. On one hand were many articles that only described surveillance systems or activities dedicated to disease control. On the other were integrated documents, such as action plans or guidelines, that did not describe implementation, results, or difficulties encountered.

Practices, and especially those of authorities in charge of risk management, must change to assign greater value to the planning strategy and the results of implementation.

To analyze the effectiveness of surveillance systems for the implementation of public health actions, a tool will need to be developed that is better adapted and integrates different methodological frameworks [93].

### Implications for future research

There is a patent need for innovative research to cope with environmental, social, or health changes (see Table 1). Innovation needed especially to contend with elimination situations, which may be the case for different parasitic diseases [18, 51, 76, 85]. Study designs should also enable constructive analysis of the data collected [94].

**Table 1:** Priority needs for future research

Improved study designs	
New tools to collect, analyze, and disseminate information (GIS, mHealth, apps)	
New tools to control vectors and pathogens because of increasing resistance to insecticides and drugs (sterile mosquitoes, Wolbachia, multiplex virus diagnoses)	
Identification of residual sources of infection for better VBD control	

Research is needed to determine relevant thresholds for early warnings of outbreaks [85] and to support the implementation of control actions [95]. Such thresholds will depend on local conditions and the surveillance system implemented. Research on evidence-based response strategies and cost-effectiveness should also be considered a priority [96]. Indeed, a decrease in vector population does not imply risk reduction, whether for dengue or for malaria [51, 72, 94, 97]. There is also a need for better knowledge about the consequences of the circulation of certain genotypes or serotypes in terms of risk [38, 62] and vectors [19, 98–100]. Tasks related to vector control have changed rapidly over the past decades, and stronger technical and communication skills are required to contend with the evolution in vector control methods and to involve communities [32]. Those conducting entomological surveillance, and more particularly larval survey, face increasing difficulties in gaining entrance to private properties [14].

Detecting the focus of residual transmission (whether breeding sites or asymptomatic patients) is challenging [51, 101]. The rapid increase of insecticide resistance in vectors underscores the need to regularly evaluate vector sensitivity to insecticides used and to develop alternative strategies such as insecticide rotations and mixtures to delay the evolution of resistance.

Arboviral diseases present specific challenges. Co-circulation of different arboviruses requires that the biological confirmation component of the human surveillance system be tailored in terms of strategy and capacity [31, 102, 103]. There is an acute need to develop a good indicator for mosquito population that should be easy to obtain or compute at the operational level, for the couple *Aedes*/arbovirus. There are also needs for evaluation of current control methods and tools, life-table studies, behavioural studies on *Aedes* mosquitoes, GIS models for forecasting dengue, etc. [70, 72]. This represents a research opportunity to better quantify this relationship and to develop tools to measure it. To optimize disease prevention, priority must be given to high-quality standardized studies that evaluate and compare methods [94].

Ultimately what is needed is to integrate vector and disease control in a single strategy [59]. Sustained coordination among governments, agencies, control programs, academia, private enterprises, and the affected communities is the foundation for the success of any future strategy [25, 26, 40, 48, 57, 60, 62, 72, 95].

### Implications for public health and/or practice

The review clearly highlighted the need for public health and research actors at all levels of the surveillance and intervention framework to be involved (see Table 2). The social burden of VBDs has to be understood and linked to outcomes such as morbidity and mortality.

**Table 2:** Implications for public health policy and/or practice

Community-based strategies are key to successful VBD control	
Intersectoral collaboration will ensure intervention sustainability and policy engagement by health and urban policy actors	
Timely release of surveillance results will facilitate prompt remedial actions for vector control	
Health education is needed to sustain public participation in vector prevention and control	
The use of protective measures such as long-lasting impregnated bed-nets and the implementation of control tools tailored to the local context (including perceptions of field workers and communities) need to be up-scaled.	

First, risk assessment should identify the main risks and threats that will need to be considered in a specific surveillance system. Surveillance objectives should be precisely defined and communicated to all the actors. Based on this assessment, surveillance and response will be planned taking into consideration available resources and gaps. This will require communication and training at different steps of the implementation process. Training has to reinforce the capacity to detect cases using appropriate tools for precise case definition, appropriate diagnostic methods, and rapid communication of laboratory results. Medical care with appropriate case management must be defined and treatments must be available. At a broader scale, the authorities must also be involved and mobilized. Preparedness and response should be formalized in a document that is regularly updated and shared among the different stakeholders. Such plans should cover early detection, epidemiological and vector surveillance, definition of a biological diagnostic strategy, guidelines for case management, vector control actions, and a social mobilization strategy. A preparedness and response plan should propose a tailored and graduated surveillance and intervention framework based on risk level. This will contribute to a better analysis of the knowledge transfer process, which has not yet been sufficiently studied [104]. Ultimately the results must be shared at different levels, not only through scientific publications, but also by wide dissemination to the field teams, medical teams, authorities, and populations. Moreover, health policy and urban policy actors need to cooperate because, while health favours development, development in turn also favours health.

## Conclusions

Overall, the results showed that the largest body of evidence concerned surveillance and intervention against arboviral diseases, mainly dengue. Our results highlighted the abundance of surveillance and control systems against VBDs around the world and gave the opportunity of a short Strengths, Weaknesses, Opportunities and Threats (SWOT) analysis (see Table 3).

**Table 3:** SWOT analysis of surveillance systems for prevention and control of VBDs in urban settings

Strengths: research-based operations and community participation; available experience and expertise	
Weaknesses: inadequate epidemiological-entomological surveillance; pathogen and insecticide resistance; poor surveillance of residual transmission; hidden breeding sites; time lag between data collection and diffusion; lack of sensitivity of surveillance system (underreporting and misdiagnosis)	
Opportunities: capacity building; research collaborations; systematic collection of pertinent data; improved municipal services; use of technologies like GIS to improve data mapping, reporting, and dissemination	
Threats: political and geographical situation; environmental and social constraints; financial constraints; emerging arboviruses; difficulty of maintaining resources for surveillance and response in contexts of elimination	

The emergence of arboviral diseases in high-income countries is drawing attention to these diseases, which no longer concern low-income countries exclusively. High-income countries are not well prepared for these threats, as has been shown in epidemics of West Nile or Zika virus, for example. Monitoring these diseases from a control perspective should put these risks on the political agenda. Such occurrences should serve as opportunities to build surveillance systems that are adapted to local contexts but based on shared rules. These rules are based on three principles: systematic collection of pertinent data; analysis of these data, and timely dissemination of results to guide interventions. All efforts must be focused on implementing these rules.

Integrated systems that concurrently target the vector in its environment, the pathogen, and the hosts—both humans and animals, if they are involved in the disease cycle—should be promoted. These initiatives are part of the One Health new paradigm which postulates that the dynamics of the diseases and the actions which determine the health of the human as well as the animal populations must be studied in their environmental context. As regularly observed but rarely implemented, the first step in such an approach should be the assessment of community knowledge, attitudes, and practice. High-level support and inter-agency cooperation are also key to the success of a control program. Broadening the scale, some studies suggested that country responses should be optimized by pooling resources and sharing experience and data. It is also time for policy-makers and the scientific community alike to pay more attention to the effects of urbanization and globalization on VBDs.

## Additional files

Additional file 1: Multilingual abstracts in the five official working languages of the United Nations. (PDF 879 kb)

Additional file 2: Search strategy. (DOCX 31 kb)

Additional file 3: Data extraction grid. (XLSX 139 kb)

Additional file 4: List of included references. (DOCX 52 kb)

## Abbreviations

ACT: Artemisinin-based Combination Therapy
AMCD: Anastasia mosquito control district
ASPCAT: Public Health Agency of Catalonia
Bti: *Bacillus thuringiensis israelensis*
CDC: Center for Disease Control
DENV: Dengue virus
DFB: Diflubenzuron
DFMP: Dengue Fever Management Plan (for north Queensland)
EANMAT: East African Network for Monitoring Antimalarial Treatment
ELISA: Enzyme-linked immunosorbent Assay
GIS: Geographic information system
HIN: Health Information Network
IEC: Information, Education, Communication
IFAT: Indirect fluorescent antibody test
IgM: Immunoglobulin M
IPT: Intermittent Preventative Treatment
IRS: Indoor residual spraying
ITN: Insecticidal treated net
lA: Larvicide application
LLIN: Long lasting insecticidal impregnated net
MCP: Mosquito Control Program
MDA: Mass-drug administration
MID: Monitoramento Inteligente da Dengue [Intelligent Dengue Monitoring System]
PCR: Polymerase chain reaction
PRDH: Puerto Rico Department of Health
PSAGE: Program for Surveillance, Alert and Response
RDT: Rapid diagnostic test
SDSS: Spatial decision support systems
SNEM: Servicio Nacional de Control de Enfermedades Transmitidas por Vectores Atrópodos (National Service for the control of VBD)
SP: Sulphadoxine-pyrimethamine
SWOT: Strengths, Weaknesses, Opportunities and Threats
VBD: Vector-borne disease

## Footnotes

1
http://www.who.int/neglected_diseases/vector_ecology/VCAG/en/

2
http://www.who.int/mediacentre/factsheets/fs387/fr/
