# Supplementary material for: Effective surveillance systems for vector-borne diseases in urban settings and translation of the data into action: a scoping review
Source: Infect Dis Poverty. 2018 Sep 3;7:99. doi: 10.1186/s40249-018-0473-9 (PMC6137924; doi:10.1186/s40249-018-0473-9)
Supplement: Supplementary file 1 — Multilingual abstracts in the five official working languages of the United Nations. (PDF 879 kb) [file 40249_2018_473_MOESM1_ESM.pdf]

Translation of the abstract into the five official working languages of the United Nations

نظم مراقبة فعالة للأمراض المنقولة بالنواقل في المناطق الحضرية وترجمة البيانات إلى إجراءات: مراجعة الفحص

فلورنس فورنيه ، فريديريك جوردان ، إيمانويل بونيه ، ستيفاني ديغروت ، فاليري ريد

الملخص
الخلفية : لا تزال الأمراض المنقولة بواسطة ناقلات الأمراض (VBDs) تمثل تهديدًا عالميًا ، مع الأمراض "القديمة" مثل الملاريا ، و "الناشئة" أو "العائدة من جديد" مثل زيكا ، بسبب الزيادة في التجارة الدولية والنمو الديموغرافي والتحضر السريع. وفي عصر العولمة هذا ، تعتبر المراقبة عنصراً رئيسياً في السيطرة على الأمراض المنقولة بالنواقل VBDs في المناطق الحضرية ، لكن المراقبة وحدها لا يمكن أن تحل المشكلة. فمن المهم مراجعة التجارب لفحص عناصر الحلول الأخرى. كانت الأهداف هي تقييم الوسائل المختلفة لمراقبة الأمراض المنقولة بالنواقل VBD في البيئات الحضرية ، وتقييم إمكاناتها لدعم إجراءات الصحة العامة ، ووصف الأدوات المستخدمة في إجراءات الصحة العامة ، والقيود التي تواجهها ، والفجوات البحثية والعملية في مجال الصحة التي ينبغي ملؤها.
المتن الرئيسي : في هذه المراجعة الاستطلاعية ، قمنا ببحث مقالات مراجعة النظراء والمطبوعات غير الرسمية بين عامي 2000 و 2016 . وتم استخدام أدوات مختلفة لتشفير البيانات واستخراجها. تم إجراء تقييم جودة لكل دراسة تمت مراجعتها ، وتم تحليل الخصائص الوصفية والبيانات المتعلقة بعملية التنفيذ وقابلية النقل في جميع الدراسات.
بعد فحص 414 نصًا كاملاً ، احتفظنا بما مجموعه 79 مقالة للمراجعة. كانت الأهداف الرئيسية للمقالات هي الأمراض الفيروسية المنقولة بالمفصليات (65.8 ٪) والملاريا (16.5 ٪). تتناسب الجوانب الإيجابية للدراسات العديدة في إطار الإدارة المتكاملة لناقلات الأمراض. يعتبر الوعي العام المفتاح لنجاح برامج مكافحة ناقلات الأمراض. يمكن أن تؤدي الدعوة والتشريع إلى تعزيز كلٍ من التمكين وبناء القدرات. ويمكن تحقيق ذلك عن طريق التعاون داخل القطاع الصحي ومع القطاعات الأخرى. هناك حاجة إلى البحوث لتطوير دراسات مصممة بشكل جيد وأدوات جديدة للمراقبة والتحكم.
الاستنتاجات : تم إبراز الحاجة إلى نظم الرصد في المناطق الحضرية في كل من البلدان النامية والمتقدمة النمو. تواجه البلدان نفس التحديات المتعلقة بالموارد البشرية والمالية والهيكلية. وتشكل هذه النتائج أيضًا دعوة لصحوة الحكومات والأوساط الأكاديمية والممولين ومنظمة الصحة العالمية من أجل تعزيز برامج المراقبة وتعزيز أبحاث الأمراض المنقولة بالنواقل VBD في البيئات الحضرية.

Translated from English version into Arabic by Free bird, proofread by Abdessalam AIT TOUIJAR, through

TRANSLATORS
WITHOUT BORDERS

评估城市环境中媒传疾病的有效监测系统，将数据转化为行动

Florence Fournet, Frédéric Jourdain, Emmanuel Bonnet, Stéphanie Degroote, Valéry Ridde

摘要
引言：由于国际贸易活动增多、人口增长、城市化进程加快，媒传疾病(VBDs) 持续对全球公众健康造成影响，如古老疾病疟疾、新发和再现疾病寨卡。在全球化时代，监测是控制城市 VBDs 的一种重要手段，但监测自身并不能解决问题。对以往经验进行回顾有助于寻找更多解决方案。本综述旨在评估城市环境中 VBD 监测的不同途径，评估其支持公共卫生行动的潜力，描述用于公共卫生行动的工具、它们所面临的制约因素，以及亟待弥合的卫生研究和行动的差距。
主要内容：我们检索了 2000 年至 2016 年间发表的同行评议文章和灰色文献，使用多种工具进行数据编码和提取。对纳入综述的每篇文献进行了质量评估，并分析其描述性特征、实施过程和可转移性的数据。

共检索到 414 篇全文文章，筛选纳入了 79 篇文章用于综述。这些文章的主要研究对象是虫媒病毒病（65.8％）和疟疾（16.5％）。许多研究的阳性结果体现了综合病媒管理框架的适应性。公众意识是病媒生物控制项目能否成功的关键。宣传和立法可以促进赋权和能力建设。这些可以通过卫生部门与其他部门合作来实现。此外，需进一步开展经精心设计的研究，开发用于监测和防制的新工具。

结论：本文强调了发展中国家和发达国家的城市环境对监测系统的需求。各国都面临着人力、财力和结构性资源的挑战。这些研究结果呼吁，各国政府、学术界、资助者和世界卫生组织需加强对控制项目的管理，并推进城市环境中的 VBD 研究。

Translated from English version into Chinese by Jin Chen, edited by Pin Yang

CHINESE CENTER FOR DISEASE CONTROL AND PREVENTION
NATIONAL INSTITUTE OF PARASITIC DISEASES

L'efficacité des systèmes de surveillance des maladies à transmission vectorielle dans les milieux urbains et la traduction des données en action : une étude exploratoire

Florence Fournet, Frédéric Jourdain, Emmanuel Bonnet, Stéphanie Degroote, Valéry Ridde

Résumé

Contexte: Qu'il s'agisse de "vieilles" maladies telles que le paludisme ou de maladies "plus récentes" comme le Zika, les maladies à transmission vectorielle (MTVs) continuent de représenter une menace pour l'ensemble de l'humanité, et ce du fait d'un accroissement du commerce international, de la croissance démographique, et du taux rapide d'urbanisation. A l'ère de la mondialisation, la surveillance est un élément-clé dans la gestion des MTVs en milieu urbain, mais celle-ci ne peut résoudre tous les problèmes. Une revue des différentes expériences permet d'examiner d'autres éléments de solution. Objectifs: évaluer les différents moyens de surveillance des MTVs, examiner leur rôle potentiel en tant que soutien des actions de santé publique, décrire les outils utilisés par les actions de santé publique, les obstacles auxquels elles font face, et les écarts à combler entre recherche et actions sanitaires.

Développement: Pour cette étude exploratoire, nous nous sommes penchés sur la littérature scientifique et autres documents publiés entre 2000 et 2016. Différents outils ont été utilisés pour l'extraction et le codage des données. Une évaluation qualitative a été réalisée pour chaque étude passée en revue; une analyse des caractéristiques descriptives et des données concernant le processus de mise en application et la transférabilité a également été menée pour toutes les études concernées.

Après avoir passé en revue 414 articles en texte intégral, nous en avons retenu 79 pour analyse. Les articles passés en revue portaient essentiellement sur les maladies causées par des arbovirus (65.8%) et sur le paludisme (16.5%). Les aspects positifs de nombreuses données s'inscrivent dans le cadre d'une prise en charge intégrée du vecteur. La sensibilisation du public est considérée comme élément-clé des programmes de lutte contre les vecteurs. Les programmes de sensibilisation et mesures législatives renforcent tout deux l'autonomisation et les capacités. Ces objectifs peuvent être atteints par une collaboration tant à l'intérieur du système de santé qu'entre le système de santé et les autres secteurs. Des recherches sont nécessaires afin de développer des études bien conçues ainsi que de nouveaux outils de surveillance et contrôle.

Conclusions: Cette étude a mis en évidence la nécessité de systèmes de surveillance en milieu urbain, dans les pays développés comme dans les pays en développement. Les pays sont confrontés aux mêmes défis posés par les ressources humaines, financières, et structurelles. Les conclusions de cette étude constituent également un signal d'alarme pour les

gouvernements, le milieu universitaire, les bailleurs de fonds et l'Organisation Mondiale de la Santé à qui il revient de renforcer les programmes de contrôle et de soutenir la recherche sur les MTVs en milieu urbain.

Translated from English version into French by Sophie N, proofread by Anne Marie, through

Эффективные системы мониторинга трансмиссивных болезней в городах и использование данных для принятия мер: аналитический обзор

Флоренсе Фурнье, Фредерик Журдейн, Эммануэль Бонне, Стефани Дегрот, Валери Ридд

Аннотация

История вопроса: Трансмиссивные болезни продолжают представлять собой глобальную угрозу. Это и “старые” болезни, такие как малярия, и “появляющиеся” или “возвращающиеся”, такие как Зика, вызванные усилением международной торговли, демографическим ростом и ускорением урбанизации. В эпоху глобализации мониторинг является ключевым элементом контроля трансмиссивных болезней в городах, но сам по себе не может решить создавшуюся проблему. Представляет интерес рассмотрение накопленного опыта с целью выделения других элементов решения проблемы. Целями исследования были оценка способов мониторинга трансмиссивных болезней в городах, анализ их потенциала для действий, связанных с народным здравоохранением, и описание средств, используемых для таковых действий, а также связанных с этим ограничений и пробелов в исследованиях и системе здравоохранения.

Основная часть: Для данного аналитического обзора был проведен поиск по рецензируемой и внеиздательской литературе, опубликованной между 2000 и 2016 годами. Для кодирования и извлечения данных использовались различные средства. Была проведена оценка каждого рассмотренного исследования, и были проанализированы описательные характеристики и данные по процессам реализации и переносимости для всех исследований.

После предварительного рассмотрения 414 полнотекстовых статей мы оставили для обзора 79 из них. Главными объектами рассмотрения в этих статьях были арбовирусные инфекции (65,8%) и малярия (16,5%). Позитивные аспекты многих исследований вписываются в рамки комплексной борьбы с переносчиками трансмиссивных болезней. Повышение уровня осведомленности общественности считается ключевым фактором для успеха программ борьбы с переносчиками инфекций. Информационно-просветительская деятельность и законодательные акты способствуют расширению возможностей и укреплению потенциала. Это может быть достигнуто путем сотрудничества с сектором здравоохранения и другими секторами. Для проведения должным образом запланированных научных работ и разработки новых средств мониторинга и контроля необходимы исследования.

Выводы: Была подчеркнута необходимость систем мониторинга в городах как в развивающихся, так и в развитых странах. Все страны сталкиваются с одинаковыми проблемами, связанными с человеческими, финансовыми и структурными ресурсами. Эти выводы также являются тревожным напоминанием членам правительств, научным кругам, инвесторам и Всемирной организации здравоохранения о необходимости укрепления программ контроля и расширения изучения трансмиссивных болезней в городах.

Translated from English version into Russian by Elena McDonnell, proofread by Natalia Potashnik, through

Sistemas eficaces de vigilancia de las enfermedades transmitidas por vectores en áreas urbanas y la transformación de información en acción: Un estudio de campo.

Florence Fournet, Frédéric Jourdain, Emmanuel Bonnet, Stéphanie Degroote, Valéry Ridde.

Resumen

Antecedentes: Las enfermedades transmitidas por vectores (ETV) siguen siendo una amenaza global, "antiguas" como el paludismo y "emergentes" o "reemergentes" como el virus Zika, debido al incremento del comercio internacional, al crecimiento demográfico y a la rápida urbanización. En esta época de globalización, la vigilancia es un elemento clave en el control de las ETV en áreas urbanas, pero no puede resolver el problema por si sola. Una análisis de las experiencias reviste interés en relación con el estudio de otros elementos resolutivos. Los objetivos fueron: evaluar los diversos métodos de vigilancia de las ETV en áreas urbanas, evaluar su potencial para apoyar las acciones de salud pública y describir qué instrumentos se usaron en aquellas acciones, los límites que tuvieron que afrontar y lo que aún hay que lograr en términos de investigación y acciones de salud.

Cuerpo principal: Esta revisión de campo implicó la búsqueda sistemática de literatura, tanto revisada como no revisada, publicada entre el 2000 y el 2016. Se utilizaron diferentes herramientas de extracción de datos para su codificación y extracción. Se evaluó la calidad de cada estudio y se analizaron las características descriptivas y los datos sobre el proceso de implementación y la transferibilidad de todos los estudios.

Después de examinar 414 artículos, seleccionamos 79 para la revisión. Los objetivos de dichos artículos eran las enfermedades por arbovirus (65.8%) y el paludismo (16.5%). Los aspectos positivos de numerosos estudios encajan en el marco de la gestión integrada de vectores. La concientización pública es considerada un factor clave para el éxito de los programas de control de vectores. Una promoción y legislación acorde pueden aumentar el empoderamiento y la creación de capacidades. Para lograrlo, es necesaria la colaboración dentro del sector salud y con otros sectores. La investigación es vital para el desarrollo de estudios bien diseñados y nuevos instrumentos de vigilancia y control.

Conclusiones: Se ha enfatizado la necesidad de un sistema de vigilancia en áreas urbanas, tanto en países en vías de desarrollo como en los desarrollados. Los países afrontan los mismos desafíos en materia de recursos humanos, estructurales y económicos. Esta conclusión también constituye una llamada de atención a los gobiernos, al mundo académico, a los patrocinadores y a la Organización Mundial de la Salud para que fortalezcan sus programas de control y mejoren sus investigaciones sobre ETV en áreas urbanas.

Translated from English version into Spanish by Maria CG, proofread by Natalia Victoria Gómez, through
