# Supplementary material for: Effective surveillance systems for vector-borne diseases in urban settings and translation of the data into action: a scoping review
Source: Infect Dis Poverty. 2018 Sep 3;7:99. doi: 10.1186/s40249-018-0473-9 (PMC6137924; doi:10.1186/s40249-018-0473-9)
Supplement: Supplementary file 2 — Search strategy. (DOCX 31 kb) [file 40249_2018_473_MOESM2_ESM.docx]

**Additional file 2: Search strategy**

**Key concepts:**

| Vector-Borne Diseases | Urban settings | Surveillance systems | Public Health actions |
| --- | --- | --- | --- |

**Associated keywords:**

| **Vector-Borne diseases** | **Urban areas** | **Surveillance** | **Public health action** |
| --- | --- | --- | --- |
| \| Aedes* \| \| --- \| \| African trypanosomiasis \| \| Alphaviruse* \| \| Alphavirus infection \| \| American trypanosomiasis \| \| Anophel* \| \| arachnid vector* \| \| Arbovirus* \| \| Arthropod vector* \| \| arthropodborne disease* \| \| Arthropod-borne virus* \| \| arthropod-transmitted zoonose* \| \| bacterial disease* \| \| Bartonellosis \| \| black flies \| \| blackflies \| \| blood-sucking arthropod* \| \| blood-sucking insect* \| \| borreliosis \| \| Chagas disease* \| \| Chikungunya* \| \| Crimean-Congo haemorrhagic fever* \| \| Culex \| \| Dengue* \| \| disease vector* \| \| encephalitis arbovirus \| \| encephalomyelitis arbovirus \| \| filariasis \| \| Flaviviruse* \| \| fleas \| \| hematophagous insect \| \| insect vector* \| \| Ixode* \| \| Japanese Encephalitis \| \| leishmaniasis \| \| lice \| \| Lyme disease* \| \| malaria vector* \| \| malaria* \| \| mosquito disease* \| \| mosquito vector* \| \| mosquito* \| \| mosquito-borne disease* \| \| Onchocerciasis \| \| parasitic disease* \| \| phlebotomus fever* \| \| plague* \| \| Q fever* \| \| Relapsing fever* \| \| Rickettsial disease* \| \| Rickettsiosis \| \| rift valley fever* \| \| river blindness \| \| sandflies \| \| sandfly fever* \| \| Sleeping sickness \| \| spotted fever* \| \| Tick-borne disease* \| \| ticks \| \| Tick-Borne Encephalitis \| \| triatom* \| \| trypanosom* \| \| tse tse flies \| \| tse tse flye \| \| Tularaemia* \| \| typhus \| \| Vector-borne disease* \| \| vector-borne pathogen* \| \| vector-borne zoonose \| \| west Nile fever \| \| West Nile Virus* \| \| Yellow Fever* \| \| Zika* \| \| aquatic snail \| \| Schistosomiasis \| \| bilharziasis \| \| communicable diseases \| \| emerging disease* \| \| neglected disease* \| \| tropical disease* \| \| zoonotic disease* \| | \| (population n4 density) \| \| --- \| \| block* \| \| borough* \| \| cities \| \| city \| \| communities \| \| community \| \| cosmopoli* \| \| district* \| \| downtown* \| \| house* \| \| megacit* \| \| megalopolis \| \| metropoli* \| \| midtown* \| \| municipal* \| \| neighborhood* \| \| neighbourhood* \| \| (public n4 infrastructure*) \| \| slum* \| \| suburb* \| \| town* \| \| Urban* \| \| village* \| \| housing \| \| countrywide \| \| nationwide \| \|  \| | \| Breteau index \| \| --- \| \| Collection \| \| Container index \| \| Entomological index \| \| (entomolog* AND threshold*) \| \| house index \| \| Larval index \| \| mobile health \| \| monitoring \| \| Notifiable disease \| \| oviposition trap \| \| ovitrap \| \| Pupal index \| \| pupal survey \| \| sentinel animal* \| \| seroprevalence \| \| serosurvey \| \| sticky trap \| \| surveillance \| \| vector density \| \| vector indices \| \| vector capacit* \| \| vector prevalence \| \| vector distribution \| \| infestation \| \| critical density \| \| morbidity \| \| mortality \| \| vector index \| | \| (evalua* n4 (health threat* OR health risk*)) \| \| --- \| \| (preparedness n4 response*) \| \| behavior change* \| \| behaviour change* \| \| blood safety \| \| community-oriented service* \| \| containment \| \| control intervention* \| \| control program* \| \| disease* control \| \| disease* management \| \| early warning system* \| \| health education \| \| (disease n4 elimination) \| \| health care \| \| health planning \| \| health promotion \| \| health resource* \| \| Inter-sectoral action* \| \| medical care \| \| policy mak* \| \| prevention \| \| (public health n4 practice*) \| \| risk assessment \| \| risk communication \| \| Risk management \| \| risk reduction \| \| participation \| \| mobilization \| \| targeting intervention* \| \| vector control \| \| planning \| \| rodent control \| \| health policy \| \| (health n4 (decision mak*)) \| \| (prevention n4 control) \| \| (education n4 public health professional) \| \| communit* awareness \| \| ((program* OR project* OR intervention* OR prevention OR control OR management) n4 (sustainab* OR routinization OR institutionalization OR effectiveness OR efficiency OR mobilization OR implication OR development)) \| \| implementation \| \| ((knowledge OR research OR evidence) n4 (transfer* OR uptak* OR disseminat* OR assimilat* OR incorporat*)) \| \| guidelines \| \| best practice* \| \| capacity building \| |

**Databases to search:**

Scientific literature: Medline (MeSH), Web of Science, Embase (EMTREE), Global Health.

Grey literature: Opengrey.

**MeSH terms/descriptors and appropriate operators for each database**

***Search for Medline***

("Aedes*"[Title/Abstract] OR "African trypanosomiasis"[Title/Abstract] OR "Alphaviruse*"[Title/Abstract] OR "Alphavirus infection"[Title/Abstract] OR "American trypanosomiasis"[Title/Abstract] OR "Anophel*"[Title/Abstract] OR "arachnid vector*"[Title/Abstract] OR "Arbovirus*"[Title/Abstract] OR "Arthropod vector*"[Title/Abstract] OR "arthropodborne disease*"[Title/Abstract] OR "Arthropod-borne virus*"[Title/Abstract] OR "arthropod-transmitted zoonose* "[Title/Abstract] OR "bacterial disease*"[Title/Abstract] OR "Bartonellosis"[Title/Abstract] OR "black flies"[Title/Abstract] OR "blackflies"[Title/Abstract] OR "blood-sucking arthropod*"[Title/Abstract] OR "blood-sucking insect*"[Title/Abstract] OR "borreliosis"[Title/Abstract] OR "Chagas disease*"[Title/Abstract] OR "Chikungunya*"[Title/Abstract] OR "Crimean-Congo haemorrhagic fever*"[Title/Abstract] OR "Culex"[Title/Abstract] OR "Dengue*"[Title/Abstract] OR "disease vector*"[Title/Abstract] OR "encephalitis arbovirus"[Title/Abstract] OR "encephalomyelitis arbovirus"[Title/Abstract] OR "filariasis"[Title/Abstract] OR "Flaviviruse*"[Title/Abstract] OR "fleas"[Title/Abstract] OR "hematophagous insect"[Title/Abstract] OR "insect vector*"[Title/Abstract] OR "Ixode*"[Title/Abstract] OR "Japanese Encephalitis"[Title/Abstract] OR "leishmaniasis"[Title/Abstract] OR "lice"[Title/Abstract] OR "Lyme disease*"[Title/Abstract] OR "malaria vector*"[Title/Abstract] OR "malaria*"[Title/Abstract] OR "mosquito disease*"[Title/Abstract] OR "mosquito vector*"[Title/Abstract] OR "mosquito*"[Title/Abstract] OR "mosquito-borne disease*"[Title/Abstract] OR "Onchocerciasis"[Title/Abstract] OR "parasitic disease*"[Title/Abstract] OR "phlebotomus fever*"[Title/Abstract] OR "plague*"[Title/Abstract] OR "Q fever*"[Title/Abstract] OR "Relapsing fever*"[Title/Abstract] OR "Rickettsial disease*"[Title/Abstract] OR "Rickettsiosis"[Title/Abstract] OR "rift valley fever*"[Title/Abstract] OR "river blindness"[Title/Abstract] OR "sandflies"[Title/Abstract] OR "sandfly fever*"[Title/Abstract] OR "Sleeping sickness"[Title/Abstract] OR "spotted fever*"[Title/Abstract] OR "Tick-borne disease*"[Title/Abstract] OR "ticks"[Title/Abstract] OR "Tick-Borne Encephalitis"[Title/Abstract] OR "triatom*"[Title/Abstract] OR "trypanosom*"[Title/Abstract] OR "tse tse flies"[Title/Abstract] OR "tse tse flye"[Title/Abstract] OR "Tularaemia*"[Title/Abstract] OR "typhus"[Title/Abstract] OR "Vector-borne disease*"[Title/Abstract] OR "vector-borne pathogen*"[Title/Abstract] OR "vector-borne zoonose"[Title/Abstract] OR "west Nile fever"[Title/Abstract] OR "West Nile Virus*"[Title/Abstract] OR "Yellow Fever*"[Title/Abstract] OR "Zika*"[Title/Abstract] OR "aquatic snail"[Title/Abstract] OR "Schistosomiasis"[Title/Abstract] OR "bilharziasis"[Title/Abstract] OR "communicable diseases"[Title/Abstract] OR "emerging disease*"[Title/Abstract] OR "neglected disease*"[Title/Abstract] OR "tropical disease*"[Title/Abstract] OR "zoonotic disease*"[Title/Abstract]) OR "Aedes"[MAJR] OR "Alphavirus"[MAJR] OR "Alphavirus Infections"[MAJR] OR "Anopheles"[MAJR] OR "Arachnid Vectors"[MAJR] OR "Arboviruses"[MAJR] OR "Arthropod Vectors"[MAJR] OR "Bartonella Infections"[MAJR] OR "Boutonneuse Fever"[MAJR] OR "Chagas Disease"[MAJR] OR "Chikungunya Fever"[MAJR] OR "Communicable Diseases"[MAJR] OR "Culex"[MAJR] OR "Culicidae"[MAJR] OR "Dengue"[MAJR] OR "Disease Vectors"[MAJR] OR "Encephalitis, Arbovirus"[MAJR] OR "Encephalitis, Japanese"[MAJR] OR "Encephalomyelitis, Equine"[MAJR] OR "Filariasis"[MAJR] OR "Flavivirus"[MAJR] OR "Hemorrhagic Fever Virus, Crimean-Congo"[MAJR] OR "Insect Vectors"[MAJR] OR "Ixodes"[MAJR] OR "Leishmaniasis"[MAJR] OR "Lyme Disease"[MAJR] OR "Malaria"[MAJR] OR "Onchocerciasis"[MAJR] OR "Onchocerciasis, Ocular"[MAJR] OR "Pediculus"[MAJR] OR "Phlebotomus Fever"[MAJR] OR "Plague"[MAJR] OR "Psychodidae"[MAJR] OR "Q Fever"[MAJR] OR "Relapsing Fever"[MAJR] OR "Rickettsia Infections"[MAJR] OR "Rift Valley Fever"[MAJR] OR "Rocky Mountain Spotted Fever"[MAJR] OR "Schistosomiasis"[MAJR] OR "Simuliidae"[MAJR] OR "Siphonaptera"[MAJR] OR "Tick-Borne Diseases"[MAJR] OR "Ticks"[MAJR] OR "Triatominae"[MAJR] OR "Trypanosomiasis"[MAJR] OR "Trypanosomiasis, African"[MAJR] OR "Tsetse Flies"[MAJR] OR "Tularemia"[MAJR] OR "West Nile Fever"[MAJR] OR "West Nile virus"[MAJR] OR "Yellow Fever"[MAJR] OR "Zika Virus"[MAJR] OR "Zika Virus Infection"[MAJR]

**AND**

("(population n4 density)"[Title/Abstract] OR "block*"[Title/Abstract] OR "borough*"[Title/Abstract] OR "cities"[Title/Abstract] OR "city"[Title/Abstract] OR "communities"[Title/Abstract] OR "community"[Title/Abstract] OR "cosmopoli*"[Title/Abstract] OR "district*"[Title/Abstract] OR "downtown*"[Title/Abstract] OR "house*"[Title/Abstract] OR "megacit*"[Title/Abstract] OR "megalopolis"[Title/Abstract] OR "metropoli*"[Title/Abstract] OR "midtown*"[Title/Abstract] OR "municipal*"[Title/Abstract] OR "neighborhood*"[Title/Abstract] OR "neighbourhood*"[Title/Abstract] OR "(public n4 infrastructure*)"[Title/Abstract] OR "slum*"[Title/Abstract] OR "suburb*"[Title/Abstract] OR "town*"[Title/Abstract] OR "Urban*"[Title/Abstract] OR "village*"[Title/Abstract] OR "housing"[Title/Abstract] OR "countrywide"[Title/Abstract] OR "nationwide"[Title/Abstract]) OR "urban population"[MAJR] OR "Urbanization"[MAJR] OR "urban health"[MAJR] OR "Cities"[MAJR] OR "City planning"[MAJR] OR "Residence characteristics"[MAJR] OR "cities"[MAJR] OR "urbanization"[MAJR] OR "city planning"[MAJR] OR "urban population"[MAJR] OR "public housing"[MAJR]

**AND**

("Breteau index"[Title/Abstract] OR "Collection"[Title/Abstract] OR "Container index"[Title/Abstract] OR "Entomological index"[Title/Abstract] OR "(entomolog*[Title/Abstract] AND threshold*)"[Title/Abstract] OR "house index"[Title/Abstract] OR "Larval index"[Title/Abstract] OR "mobile health"[Title/Abstract] OR "monitoring"[Title/Abstract] OR "Notifiable disease"[Title/Abstract] OR "oviposition trap"[Title/Abstract] OR "ovitrap"[Title/Abstract] OR "Pupal index"[Title/Abstract] OR "pupal survey"[Title/Abstract] OR "sentinel animal*"[Title/Abstract] OR "seroprevalence"[Title/Abstract] OR "serosurvey"[Title/Abstract] OR "sticky trap"[Title/Abstract] OR "surveillance"[Title/Abstract] OR "vector density"[Title/Abstract] OR "vector indices"[Title/Abstract] OR "vector capacit*"[Title/Abstract] OR "vector prevalence"[Title/Abstract] OR "vector distribution"[Title/Abstract] OR "infestation"[Title/Abstract] OR "critical density"[Title/Abstract] OR "morbidity"[Title/Abstract] OR "mortality"[Title/Abstract] OR "vector index"[Title/Abstract]) OR "Epidemiology"[MAJR] OR "Environmental Monitoring"[MAJR] OR "Epidemiological Monitoring"[MAJR] OR "Population Surveillance"[MAJR] OR "Sentinel Surveillance"[MAJR] OR "Public Health Surveillance"[MAJR] OR "Telemedicine"[MAJR] OR "Disease Notification"[MAJR] OR "Epidemiologic Methods"[MAJR] OR "data collection"[MAJR] OR "Disease Notification"[MAJR] OR "Seroepidemiologic Studies"[MAJR]

**AND**

("(evalua* n4 (health threat*[Title/Abstract] OR health risk*))"[Title/Abstract] OR "(preparedness n4 response*)"[Title/Abstract] OR "behavior change*"[Title/Abstract] OR "behaviour change*"[Title/Abstract] OR "blood safety"[Title/Abstract] OR "community-oriented service*"[Title/Abstract] OR "containment"[Title/Abstract] OR "control intervention*"[Title/Abstract] OR "control program*"[Title/Abstract] OR "disease* control"[Title/Abstract] OR "disease* management"[Title/Abstract] OR "early warning system*"[Title/Abstract] OR "health education"[Title/Abstract] OR "(disease n4 elimination)"[Title/Abstract] OR "health care"[Title/Abstract] OR "health planning"[Title/Abstract] OR "health promotion"[Title/Abstract] OR "health resource*"[Title/Abstract] OR "Inter-sectoral action*"[Title/Abstract] OR "medical care"[Title/Abstract] OR "policy mak*"[Title/Abstract] OR "prevention "[Title/Abstract] OR "(public health n4 practice*)"[Title/Abstract] OR "risk assessment"[Title/Abstract] OR "risk communication"[Title/Abstract] OR "Risk management"[Title/Abstract] OR "risk reduction"[Title/Abstract] OR "participation"[Title/Abstract] OR "mobilization"[Title/Abstract] OR "targeting intervention*"[Title/Abstract] OR "vector control"[Title/Abstract] OR "planning"[Title/Abstract] OR "rodent control"[Title/Abstract] OR "health policy"[Title/Abstract] OR "(health n4 (decision mak*))"[Title/Abstract] OR "(prevention n4 control)"[Title/Abstract] OR "(education n4 public health professional)"[Title/Abstract] OR "communit* awareness"[Title/Abstract] OR "((program*[Title/Abstract] OR project*[Title/Abstract] OR intervention*[Title/Abstract] OR prevention[Title/Abstract] OR control[Title/Abstract] OR management) n4 (sustainab*[Title/Abstract] OR routinization[Title/Abstract] OR institutionalization[Title/Abstract] OR effectiveness[Title/Abstract] OR efficiency[Title/Abstract] OR mobilization[Title/Abstract] OR implication[Title/Abstract] OR development))"[Title/Abstract] OR "implementation"[Title/Abstract] OR "((knowledge[Title/Abstract] OR research[Title/Abstract] OR evidence) n4 (transfer*[Title/Abstract] OR uptak*[Title/Abstract] OR disseminat*[Title/Abstract] OR assimilat*[Title/Abstract] OR incorporat*))"[Title/Abstract] OR "guidelines"[Title/Abstract] OR "best practice*"[Title/Abstract] OR "capacity building"[Title/Abstract]) OR "Public health"[MAJR] OR "Health Impact Assessment"[MAJR] OR "Communicable Disease Control"[MAJR] OR "Infection Control"[MAJR] OR "Pest Control"[MAJR] OR "Insect Control"[MAJR] OR "Mosquito Control"[MAJR] OR "Rodent Control"[MAJR] OR "Tick Control"[MAJR] OR "Sanitation"[MAJR] OR "Risk Management"[MAJR] OR "Blood Safety"[MAJR] OR "Consumer Participation"[MAJR] OR "Containment of Biohazards"[MAJR] OR "Education"[MAJR] OR "Education, Public Health Professional"[MAJR] OR "Health Education"[MAJR] OR "Community Health Services"[MAJR] OR "Health Planning"[MAJR] OR "Health Promotion"[MAJR] OR "Decision Support Techniques"[MAJR] OR "Health Policy"[MAJR] OR "Policy Making"[MAJR] OR "prevention n4 control"[MAJR] OR "Primary Prevention"[MAJR] OR "Public Health Practice"[MAJR] OR "Risk Assessment"[MAJR] OR "Health Communication"[MAJR] OR "Capacity Building"[MAJR] OR "Practice Guidelines as Topic"[MAJR]

Results: 4,221

***Search for Web of SCience***

TS="Aedes*" OR TS="African trypanosomiasis" OR TS="Alphaviruse*" OR TS="Alphavirus infection" OR TS="American trypanosomiasis" OR TS="Anophel*" OR TS="arachnid vector*" OR TS="Arbovirus*" OR TS="Arthropod vector*" OR TS="arthropodborne disease*" OR TS="Arthropod-borne virus*" OR TS="arthropod-transmitted zoonose* " OR TS="bacterial disease*" OR TS="Bartonellosis" OR TS="black flies" OR TS="blackflies" OR TS="blood-sucking arthropod*" OR TS="blood-sucking insect*" OR TS="borreliosis" OR TS="Chagas disease*" OR TS="Chikungunya*" OR TS="Crimean-Congo haemorrhagic fever*" OR TS="Culex" OR TS="Dengue*" OR TS="disease vector*" OR TS="encephalitis arbovirus" OR TS="encephalomyelitis arbovirus" OR TS="filariasis" OR TS="Flaviviruse*" OR TS="fleas" OR TS="hematophagous insect" OR TS="insect vector*" OR TS="Ixode*" OR TS="Japanese Encephalitis" OR TS="leishmaniasis" OR TS="lice" OR TS="Lyme disease*" OR TS="malaria vector*" OR TS="malaria*" OR TS="mosquito disease*" OR TS="mosquito vector*" OR TS="mosquito*" OR TS="mosquito-borne disease*" OR TS="Onchocerciasis" OR TS="parasitic disease*" OR TS="phlebotomus fever*" OR TS="plague*" OR TS="Q fever*" OR TS="Relapsing fever*" OR TS="Rickettsial disease*" OR TS="Rickettsiosis" OR TS="rift valley fever*" OR TS="river blindness" OR TS="sandflies" OR TS="sandfly fever*" OR TS="Sleeping sickness" OR TS="spotted fever*" OR TS="Tick-borne disease*" OR TS="ticks" OR TS="Tick-Borne Encephalitis" OR TS="triatom*" OR TS="trypanosom*" OR TS="tse tse flies" OR TS="tse tse flye" OR TS="Tularaemia*" OR TS="typhus" OR TS="Vector-borne disease*" OR TS="vector-borne pathogen*" OR TS="vector-borne zoonose" OR TS="west Nile fever" OR TS="West Nile Virus*" OR TS="Yellow Fever*" OR TS="Zika*" OR TS="aquatic snail" OR TS="Schistosomiasis" OR TS="bilharziasis" OR TS="communicable diseases" OR TS="emerging disease*" OR TS="neglected disease*" OR TS="tropical disease*" OR TS="zoonotic disease*"

**AND**

TS=(population NEAR/4 density) OR TS=block* OR TS=borough* OR TS=cities OR TS=city OR TS=communities OR TS=community OR TS=cosmopoli* OR TS=district* OR TS=downtown* OR TS=house* OR TS=megacit* OR TS=megalopolis OR TS=metropoli* OR TS=midtown* OR TS=municipal* OR TS=neighborhood* OR TS=neighbourhood* OR TS=(public NEAR/4 infrastructure*) OR TS=slum* OR TS=suburb* OR TS=town* OR TS=Urban* OR TS=village* OR TS=housing OR TS=countrywide OR TS=nationwide

**AND**

TS="Breteau index" OR TS="Collection" OR TS="Container index" OR TS="Entomological index" OR TS="(entomolog* AND threshold*)" OR TS="house index" OR TS="Larval index" OR TS="mobile health" OR TS="monitoring" OR TS="Notifiable disease" OR TS="oviposition trap" OR TS="ovitrap" OR TS="Pupal index" OR TS="pupal survey" OR TS="sentinel animal*" OR TS="seroprevalence" OR TS="serosurvey" OR TS="sticky trap" OR TS="surveillance" OR TS="vector density" OR TS="vector indices" OR TS="vector capacit*" OR TS="vector prevalence" OR TS="vector distribution" OR TS="infestation" OR TS="critical density" OR TS="morbidity" OR TS="mortality" OR TS="vector index"

**AND**

TS="(evalua* AND (health threat* OR health risk*))" OR TS="(preparedness AND response*)" OR TS="behavior change*" OR TS="behaviour change*" OR TS="blood safety" OR TS="community-oriented service*" OR TS="containment" OR TS="control intervention*" OR TS="control program*" OR TS="disease* control" OR TS="disease* management" OR TS="early warning system*" OR TS="health education" OR TS="(disease AND elimination)" OR TS="health care" OR TS="health planning" OR TS="health promotion" OR TS="health resource*" OR TS="Inter-sectoral action*" OR TS="medical care" OR TS="policy mak*" OR TS="prevention " OR TS="(public health AND practice*)" OR TS="risk assessment" OR TS="risk communication" OR TS="Risk management" OR TS="risk reduction" OR TS="participation" OR TS="mobilization" OR TS="targeting intervention*" OR TS="vector control" OR TS="planning" OR TS="rodent control" OR TS="health policy" OR TS="(health AND (decision mak*))" OR TS="(prevention AND control)" OR TS="(education AND public health professional)" OR TS="communit* awareness" OR TS="((program* OR project* OR intervention* OR prevention OR control OR management) AND (sustainab* OR routinization OR institutionalization OR effectiveness OR efficiency OR mobilization OR implication OR development))" OR TS="implementation" OR TS="((knowledge OR research OR evidence) AND (transfer* OR uptak* OR disseminat* OR assimilat* OR incorporat*))" OR TS="guidelines" OR TS="best practice*" OR TS="capacity building"

Results: 5,858

***Search for Embase***

(evalua*.ti,ab,kw. ADJ4 (health threat*.ti,ab,kw. OR health risk*.ti,ab,kw.)) or (preparedness.ti,ab,kw. ADJ4 response*.ti,ab,kw.) or behavior change*.ti,ab,kw. or behaviour change*.ti,ab,kw. or blood safety.ti,ab,kw. or community-oriented service*.ti,ab,kw. or containment.ti,ab,kw. or control intervention*.ti,ab,kw. or control program*.ti,ab,kw. or disease* control.ti,ab,kw. or disease* management.ti,ab,kw. or early warning system*.ti,ab,kw. or health education.ti,ab,kw. or (disease.ti,ab,kw. ADJ4 elimination.ti,ab,kw.) or health care.ti,ab,kw. or health planning.ti,ab,kw. or health promotion.ti,ab,kw. or health resource*.ti,ab,kw. or Inter-sectoral action*.ti,ab,kw. or medical care.ti,ab,kw. or policy mak*.ti,ab,kw. or prevention .ti,ab,kw. or (public health.ti,ab,kw. ADJ4 practice*.ti,ab,kw.) or risk assessment.ti,ab,kw. or risk communication.ti,ab,kw. or Risk management.ti,ab,kw. or risk reduction.ti,ab,kw. or participation.ti,ab,kw. or mobilization.ti,ab,kw. or targeting intervention*.ti,ab,kw. or vector control.ti,ab,kw. or planning.ti,ab,kw. or rodent control.ti,ab,kw. or health policy.ti,ab,kw. or (health.ti,ab,kw. ADJ4 decision mak*.ti,ab,kw.) or (prevention.ti,ab,kw. ADJ4 control.ti,ab,kw.) or (education.ti,ab,kw. ADJ4 public health professional*.ti,ab,kw.) or communit* awareness.ti,ab,kw. or ((program*.ti,ab,kw. OR project*.ti,ab,kw. OR intervention*.ti,ab,kw. OR prevention.ti,ab,kw. OR control.ti,ab,kw. OR management.ti,ab,kw.) ADJ4 (sustainab*.ti,ab,kw. OR routinization.ti,ab,kw. OR institutionalization.ti,ab,kw. OR effectiveness.ti,ab,kw. OR efficiency.ti,ab,kw. OR mobilization.ti,ab,kw. OR implication.ti,ab,kw. OR development.ti,ab,kw.)) or implementation.ti,ab,kw. or ((knowledge.ti,ab,kw. OR research.ti,ab,kw. OR evidence.ti,ab,kw.) ADJ4 (transfer*.ti,ab,kw. OR uptak*.ti,ab,kw. OR disseminat*.ti,ab,kw. OR assimilat*.ti,ab,kw. OR incorporat*.ti,ab,kw.)) or guidelines.ti,ab,kw. or best practice*.ti,ab,kw. or capacity building.ti,ab,kw. OR behavior change.sh or blood safety.sh or communicable disease control.sh or community care.sh or crisis intervention.sh or decision making.sh or disease control.sh or early intervention.sh or environmental planning.sh or environmental sanitation.sh or health behavior.sh or health care.sh or health care policy.sh or health education.sh or health impact assessment.sh or health program.sh or health promotion.sh or health service.sh or hospital policy.sh or infection control.sh or mobilization.sh or organization and management.sh or pateint care planning.sh or patient care.sh or practice guideline.sh or prevention.sh or prophylaxis.sh or Public health.sh or public health.sh or public health services.sh or risk assessment.sh or Risk management.sh or rodent control.sh or sanitation.sh or social participation.sh or vector control.sh

Breteau index.ti,ab,kw. or Collection.ti,ab,kw. or Container index.ti,ab,kw. or Entomological index.ti,ab,kw. or (entomolog* AND threshold*).ti,ab,kw. or house index.ti,ab,kw. or Larval index.ti,ab,kw. or mobile health.ti,ab,kw. or monitoring.ti,ab,kw. or Notifiable disease.ti,ab,kw. or oviposition trap.ti,ab,kw. or ovitrap.ti,ab,kw. or Pupal index.ti,ab,kw. or pupal survey.ti,ab,kw. or sentinel animal*.ti,ab,kw. or seroprevalence.ti,ab,kw. or serosurvey.ti,ab,kw. or sticky trap.ti,ab,kw. or surveillance.ti,ab,kw. or vector density.ti,ab,kw. or vector indices.ti,ab,kw. or vector capacit*.ti,ab,kw. or vector prevalence.ti,ab,kw. or vector distribution.ti,ab,kw. or infestation.ti,ab,kw. or critical density.ti,ab,kw. or morbidity.ti,ab,kw. or mortality.ti,ab,kw. or vector index.ti,ab,kw. OR Disease Notification.sh or disease surveillance.sh or disease surveillance.sh or Environmental Monitoring.sh or Epidemiological Monitoring.sh or Epidemiology.sh or health survey.sh or morbidity.sh or mortality.sh or sentinel surveillance.sh or seroprevalence.sh

(population.ti,ab,kw. ADJ4 density.ti,ab,kw.) or block*.ti,ab,kw. or borough*.ti,ab,kw. or cities.ti,ab,kw. or city.ti,ab,kw. or communities.ti,ab,kw. or community.ti,ab,kw. or cosmopoli*.ti,ab,kw. or district*.ti,ab,kw. or downtown*.ti,ab,kw. or house*.ti,ab,kw. or megacit*.ti,ab,kw. or megalopolis.ti,ab,kw. or metropoli*.ti,ab,kw. or midtown*.ti,ab,kw. or municipal*.ti,ab,kw. or neighborhood*.ti,ab,kw. or neighbourhood*.ti,ab,kw. or (public.ti,ab,kw. ADJ4 infrastructure*.ti,ab,kw.) or slum*.ti,ab,kw. or suburb*.ti,ab,kw. or town*.ti,ab,kw. or Urban*.ti,ab,kw. or village*.ti,ab,kw. or housing.ti,ab,kw. or countrywide.ti,ab,kw. or nationwide.ti,ab,kw. Or urban population.sh or Urbanization.sh or city.sh or community.sh or City planning.sh or housing.sh or urabn area.sh or neighborhood.sh

Aedes*.ti,ab,kw. or African trypanosomiasis.ti,ab,kw. or Alphaviruse*.ti,ab,kw. or Alphavirus infection.ti,ab,kw. or American trypanosomiasis.ti,ab,kw. or Anophel*.ti,ab,kw. or arachnid vector*.ti,ab,kw. or Arbovirus*.ti,ab,kw. or Arthropod vector*.ti,ab,kw. or arthropodborne disease*.ti,ab,kw. or Arthropod-borne virus*.ti,ab,kw. or arthropod-transmitted zoonose* .ti,ab,kw. or bacterial disease*.ti,ab,kw. or Bartonellosis.ti,ab,kw. or black flies.ti,ab,kw. or blackflies.ti,ab,kw. or blood-sucking arthropod*.ti,ab,kw. or blood-sucking insect*.ti,ab,kw. or borreliosis.ti,ab,kw. or Chagas disease*.ti,ab,kw. or Chikungunya*.ti,ab,kw. or Crimean-Congo haemorrhagic fever*.ti,ab,kw. or Culex.ti,ab,kw. or Dengue*.ti,ab,kw. or disease vector*.ti,ab,kw. or encephalitis arbovirus.ti,ab,kw. or encephalomyelitis arbovirus.ti,ab,kw. or filariasis.ti,ab,kw. or Flaviviruse*.ti,ab,kw. or fleas.ti,ab,kw. or hematophagous insect.ti,ab,kw. or insect vector*.ti,ab,kw. or Ixode*.ti,ab,kw. or Japanese Encephalitis.ti,ab,kw. Or leishmaniasis.ti,ab,kw. or lice.ti,ab,kw. or Lyme disease*.ti,ab,kw. or malaria vector*.ti,ab,kw. or malaria*.ti,ab,kw. or mosquito disease*.ti,ab,kw. or mosquito vector*.ti,ab,kw. or mosquito*.ti,ab,kw. or mosquito-borne disease*.ti,ab,kw. or Onchocerciasis.ti,ab,kw. or parasitic disease*.ti,ab,kw. or phlebotomus fever*.ti,ab,kw. or plague*.ti,ab,kw. or Q fever*.ti,ab,kw. or Relapsing fever*.ti,ab,kw. or Rickettsial disease*.ti,ab,kw. or Rickettsiosis.ti,ab,kw. or rift valley fever*.ti,ab,kw. or river blindness.ti,ab,kw. or sandflies.ti,ab,kw. or sandfly fever*.ti,ab,kw. or Sleeping sickness.ti,ab,kw. or spotted fever*.ti,ab,kw. or Tick-borne disease*.ti,ab,kw. or ticks.ti,ab,kw. or Tick-Borne Encephalitis.ti,ab,kw. or triatom*.ti,ab,kw. or trypanosom*.ti,ab,kw. or tse tse flies.ti,ab,kw. or tse tse flye.ti,ab,kw. or Tularaemia*.ti,ab,kw. or typhus.ti,ab,kw. or Vector-borne disease*.ti,ab,kw. or vector-borne pathogen*.ti,ab,kw. or vector-borne zoonose.ti,ab,kw. or west Nile fever.ti,ab,kw. Or West Nile Virus*.ti,ab,kw. or Yellow Fever*.ti,ab,kw. or Zika*.ti,ab,kw. or aquatic snail.ti,ab,kw. or Schistosomiasis.ti,ab,kw. or bilharziasis.ti,ab,kw. or communicable diseases.ti,ab,kw. or emerging disease*.ti,ab,kw. or neglected disease*.ti,ab,kw. or tropical disease*.ti,ab,kw. or zoonotic disease*.ti,ab,kw. Or mosquito.sh or African trypanosomiasis.sh or alphavirus.sh or virus vector.sh or parasite vector.sh or arbovirus.sh or Bartonellosis.sh or simuliidae.sh or Borrelia infection.sh or Chagas disease.sh or chikungunya.sh or Chikungunya virus.sh or Crimean-Congo haemorrhagic fever virus.sh or dengue.sh or disease carrier.sh or epidemic encephalitis.sh or filariasis.sh or flea.sh or tick.sh or tick borne disease.sh or ixodes.sh or flavivirus.sh or leishmaniasis.sh or louse.sh or malaria.sh or sandfly fever.sh or plague.sh or Rickettsiosis.sh or Phlebotominae.sh or African trypanosomiasis.sh or Onchocerciasis.sh or triatominae.sh or glossinidae.sh or tularemia.sh or typhus.sh or Schistosomiasis.sh

Results: 4,158

***Search for Global Health***

Aedes$.ti,ab. OR African trypanosomiasis.ti,ab. OR Alphaviruse$.ti,ab. OR Alphavirus infection.ti,ab. OR American trypanosomiasis.ti,ab. OR Anophel$.ti,ab. OR arachnid vector$.ti,ab. OR Arbovirus$.ti,ab. OR Arthropod vector$.ti,ab. OR arthropodborne disease$.ti,ab. OR Arthropod-borne virus$.ti,ab. OR arthropod-transmitted zoonose$ .ti,ab. OR bacterial disease$.ti,ab. OR Bartonellosis.ti,ab. OR black flies.ti,ab. OR blackflies.ti,ab. OR blood-sucking arthropod$.ti,ab. OR blood-sucking insect$.ti,ab. OR borreliosis.ti,ab. OR Chagas disease$.ti,ab. OR Chikungunya$.ti,ab. OR Crimean-Congo haemorrhagic fever$.ti,ab. OR Culex.ti,ab. OR Dengue$.ti,ab. OR disease vector$.ti,ab. OR encephalitis arbovirus.ti,ab. OR encephalomyelitis arbovirus.ti,ab. OR filariasis.ti,ab. OR Flaviviruse$.ti,ab. OR fleas.ti,ab. OR hematophagous insect.ti,ab. OR insect vector$.ti,ab. OR Ixode$.ti,ab. OR Japanese Encephalitis.ti,ab. OR leishmaniasis.ti,ab. OR lice.ti,ab. OR Lyme disease$.ti,ab. OR malaria vector$.ti,ab. OR malaria$.ti,ab. OR mosquito disease$.ti,ab. OR mosquito vector$.ti,ab. OR mosquito$.ti,ab. OR mosquito-borne disease$.ti,ab. OR Onchocerciasis.ti,ab. OR parasitic disease$.ti,ab. OR phlebotomus fever$.ti,ab. OR plague$.ti,ab. OR Q fever$.ti,ab. OR Relapsing fever$.ti,ab. OR Rickettsial disease$.ti,ab. OR Rickettsiosis.ti,ab. OR rift valley fever$.ti,ab. OR river blindness.ti,ab. OR sandflies.ti,ab. OR sandfly fever$.ti,ab. OR Sleeping sickness.ti,ab. OR spotted fever$.ti,ab. OR Tick-borne disease$.ti,ab. OR ticks.ti,ab. OR Tick-Borne Encephalitis.ti,ab. OR triatom$.ti,ab. OR trypanosom$.ti,ab. OR tse tse flies.ti,ab. OR tse tse flye.ti,ab. OR Tularaemia$.ti,ab. OR typhus.ti,ab. OR Vector-borne disease$.ti,ab. OR vector-borne pathogen$.ti,ab. OR vector-borne zoonose.ti,ab. OR west Nile fever.ti,ab. OR West Nile Virus$.ti,ab. OR Yellow Fever$.ti,ab. OR Zika$.ti,ab. OR aquatic snail.ti,ab. OR Schistosomiasis.ti,ab. OR bilharziasis.ti,ab. OR communicable diseases.ti,ab. OR emerging disease$.ti,ab. OR neglected disease$.ti,ab. OR tropical disease$.ti,ab. OR zoonotic disease$.ti,ab. OR Aedes.sh. OR African trypanosomiasis.sh. OR Alphavirus.sh. OR Anopheles.sh. OR arboviruses.sh. OR Chagas' disease.sh. OR Chikungunya virus.sh. OR Crimean-Congo haemorrhagic fever virus.sh. OR Culex.sh. OR Culicidae.sh. OR dengue.sh. OR disease vectors.sh. OR equine encephalomyelitis virus.sh. OR filariasis.sh. OR Flavivirus.sh. OR Glossina.sh. OR Ixodes.sh. OR Japanese encephalitis.sh. OR leishmaniasis.sh. OR Lyme disease.sh. OR malaria.sh. OR Metastigmata.sh. OR mosquito-borne diseases.sh. OR onchocerciasis.sh. OR Phthiraptera.sh. OR plague.sh. OR Psychodidae.sh. OR Q fever.sh. OR relapsing fever.sh. OR rickettsial diseases.sh. OR Rift Valley fever.sh. OR sandfly fever.sh. OR schistosomiasis.sh. OR Simuliidae.sh. OR Siphonaptera.sh. OR spotted fever.sh. OR tickborne diseases.sh. OR tickborne encephalitis.sh. OR Triatominae.sh. OR tularemia.sh. OR typhus fevers.sh. OR vector-borne diseases.sh. OR vectors.sh. OR West Nile Fever.sh. OR West Nile virus.sh. OR yellow fever.sh. OR Yellow fever virus.sh. OR Zika virus.sh.

**AND**

(population.ti,ab. ADJ4 density.ti,ab.) OR block$.ti,ab. OR borough$.ti,ab. OR cities.ti,ab. OR city.ti,ab. OR communities.ti,ab. OR community.ti,ab. OR cosmopoli$.ti,ab. OR district$.ti,ab. OR downtown$.ti,ab. OR house$.ti,ab. OR megacit$.ti,ab. OR megalopolis.ti,ab. OR metropoli$.ti,ab. OR midtown$.ti,ab. OR municipal$.ti,ab. OR neighborhood$.ti,ab. OR neighbourhood$.ti,ab. OR (public.ti,ab. ADJ4 infrastructure$.ti,ab.) OR slum$.ti,ab. OR suburb$.ti,ab. OR town$.ti,ab. OR Urban$.ti,ab. OR village$.ti,ab. OR housing.ti,ab. OR countrywide.ti,ab. OR nationwide.ti,ab. OR cities.sh. OR communities.sh. OR housing.sh. OR neighbourhoods.sh. OR population density.sh. OR settlement.sh. OR settlement patterns.sh. OR suburban areas.sh. OR towns.sh. OR urban areas.sh. OR urban environment.sh. OR urban population.sh. OR urban sites.sh. OR urbanization.sh.

**AND**

Breteau index.ti,ab. OR Collection.ti,ab. OR Container index.ti,ab. OR Entomological index.ti,ab. OR (entomolog$.ti,ab. ADJ2 threshold$.ti,ab.) OR house index.ti,ab. OR Larval index.ti,ab. OR mobile health.ti,ab. OR monitoring.ti,ab. OR Notifiable disease.ti,ab. OR oviposition trap.ti,ab. OR ovitrap.ti,ab. OR Pupal index.ti,ab. OR pupal survey.ti,ab. OR sentinel animal$.ti,ab. OR seroprevalence.ti,ab. OR serosurvey.ti,ab. OR sticky trap.ti,ab. OR surveillance.ti,ab. OR vector density.ti,ab. OR vector indices.ti,ab. OR vector capacit$.ti,ab. OR vector prevalence.ti,ab. OR vector distribution.ti,ab. OR infestation.ti,ab. OR critical density.ti,ab. OR morbidity.ti,ab. OR mortality.ti,ab. OR vector index.ti,ab. OR data collection.sh. OR disease distribution.sh. OR disease prevalence.sh. OR disease statistics.sh. OR disease surveys.sh. OR epidemiological surveys.sh. OR epidemiology.sh. OR monitoring.sh. OR sentinel surveillance.sh. OR serological surveys.sh. OR surveillance.sh.

**AND**

(evalua$.ti,ab. ADJ4 (health threat$.ti,ab. OR health risk$.ti,ab.)) OR (preparedness.ti,ab. ADJ4 response$.ti,ab.) OR behavior change$.ti,ab. OR behaviour change$.ti,ab. OR blood safety.ti,ab. OR community-oriented service$.ti,ab. OR containment.ti,ab. OR control intervention$.ti,ab. OR control program$.ti,ab. OR disease$ control.ti,ab. OR disease$ management.ti,ab. OR early warning system$.ti,ab. OR health education.ti,ab. OR (disease.ti,ab. ADJ4 elimination.ti,ab.) OR health care.ti,ab. OR health planning.ti,ab. OR health promotion.ti,ab. OR health resource$.ti,ab. OR Inter-sectoral action$.ti,ab. OR medical care.ti,ab. OR policy mak$.ti,ab. OR prevention .ti,ab. OR (public health.ti,ab. ADJ4 practice$.ti,ab.) OR risk assessment.ti,ab. OR risk communication.ti,ab. OR Risk management.ti,ab. OR risk reduction.ti,ab. OR participation.ti,ab. OR mobilization.ti,ab. OR targeting intervention$.ti,ab. OR vector control.ti,ab. OR planning.ti,ab. OR rodent control.ti,ab. OR health policy.ti,ab. OR (health.ti,ab. ADJ4 decision mak$.ti,ab.) OR (prevention.ti,ab. ADJ4 control.ti,ab.) OR (education.ti,ab. ADJ4 public health professional$.ti,ab.) OR communit$ awareness.ti,ab. OR ((program$.ti,ab. OR project$.ti,ab. OR intervention$.ti,ab. OR prevention.ti,ab. OR control.ti,ab. OR management.ti,ab.) ADJ4 (sustainab$.ti,ab. OR routinization.ti,ab. OR institutionalization.ti,ab. OR effectiveness.ti,ab. OR efficiency.ti,ab. OR mobilization.ti,ab. OR implication.ti,ab. OR development.ti,ab.)) OR implementation.ti,ab. OR ((knowledge.ti,ab. OR research.ti,ab. OR evidence.ti,ab.) ADJ4 (transfer$.ti,ab. OR uptak$.ti,ab. OR disseminat$.ti,ab. OR assimilat$.ti,ab. OR incorporat$.ti,ab.)) OR guidelines.ti,ab. OR best practice$.ti,ab. OR capacity building.ti,ab. OR community involvement.sh. OR decision making.sh. OR decision support systems.sh. OR disease control.sh. OR health behaviour.sh. OR health care.sh. OR health impact assessment.sh. OR health policy.sh. OR health programmes.sh. OR health promotion.sh. OR health protection.sh. OR health services.sh. OR insect control.sh. OR mollusc control.sh. OR mollusc control.sh. OR patient care.sh. OR patient care.sh. OR pest control.sh. OR pest control.sh. OR public health.sh. OR public health services.sh. OR risk analysis.sh. OR risk assessment.sh. OR rodent control.sh. OR sanitation.sh. OR social participation.sh. OR vector control.sh.

Results: 5,970

***Search for OpenGrey***

("Aedes*" OR "African trypanosomiasis" OR "Alphaviruse*" OR "Alphavirus infection" OR "American trypanosomiasis" OR "Anophel*" OR "arachnid vector*" OR "Arbovirus*" OR "Arthropod vector*" OR "arthropodborne disease*" OR "Arthropod-borne virus*" OR "arthropod-transmitted zoonose* " OR "bacterial disease*" OR "Bartonellosis" OR "black flies" OR "blackflies" OR "blood-sucking arthropod*" OR "blood-sucking insect*" OR "borreliosis" OR "Chagas disease*" OR "Chikungunya*" OR "Crimean-Congo haemorrhagic fever*" OR "Culex" OR "Dengue*" OR "disease vector*" OR "encephalitis arbovirus" OR "encephalomyelitis arbovirus" OR "filariasis" OR "Flaviviruse*" OR "fleas" OR "hematophagous insect" OR "insect vector*" OR "Ixode*" OR "Japanese Encephalitis" OR "leishmaniasis" OR "lice" OR "Lyme disease*" OR "malaria vector*" OR "malaria*" OR "mosquito disease*" OR "mosquito vector*" OR "mosquito*" OR "mosquito-borne disease*" OR "Onchocerciasis" OR "parasitic disease*" OR "phlebotomus fever*" OR "plague*" OR "Q fever*" OR "Relapsing fever*" OR "Rickettsial disease*" OR "Rickettsiosis" OR "rift valley fever*" OR "river blindness" OR "sandflies" OR "sandfly fever*" OR "Sleeping sickness" OR "spotted fever*" OR "Tick-borne disease*" OR "ticks" OR "Tick-Borne Encephalitis" OR "triatom*" OR "trypanosom*" OR "tse tse flies" OR "tse tse flye" OR "Tularaemia*" OR "typhus" OR "Vector-borne disease*" OR "vector-borne pathogen*" OR "vector-borne zoonose" OR "west Nile fever" OR "West Nile Virus*" OR "Yellow Fever*" OR "Zika*" OR "aquatic snail" OR "Schistosomiasis" OR "bilharziasis") **AND** ((population NEAR/4 density) OR block* OR borough* OR cities OR city OR communities OR community OR cosmopoli* OR district* OR downtown* OR house* OR megacit* OR megalopolis OR metropoli* OR midtown* OR municipal* OR neighborhood* OR neighbourhood* OR (public NEAR/4 infrastructure*) OR slum* OR suburb* OR town* OR Urban* OR village* OR housing OR countrywide OR nationwide) **AND** ("Breteau index" OR "Collection" OR "Container index" OR "Entomological index" OR "(entomolog* AND threshold*)" OR "house index" OR "Larval index" OR "mobile health" OR "monitoring" OR "Notifiable disease" OR "oviposition trap" OR "ovitrap" OR "Pupal index" OR "pupal survey" OR "sentinel animal*" OR "seroprevalence" OR "serosurvey" OR "sticky trap" OR "surveillance" OR "vector density" OR "vector indices" OR "vector capacit*" OR "vector prevalence" OR "vector distribution" OR "infestation" OR "critical density" OR "morbidity" OR "mortality" OR "vector index")

Results: 16

**Synthesis:**

| **Keywords / Descriptors** | **Number of documents found** |
| --- | --- |
| **Medline** | **4,221** |
| **Web of Science Core Collection** | **5,858** |
| **Embase** | **4,158** |
| **Global Health** | **5,970** |
| **Grey literature (Open grey)** | **16** |

Records identified through database searching: 20,207

Records after duplicates removed (without grey litterature) : 13,764
