# Supplementary material for: Effective surveillance systems for vector-borne diseases in urban settings and translation of the data into action: a scoping review
Source: Infect Dis Poverty. 2018 Sep 3;7:99. doi: 10.1186/s40249-018-0473-9 (PMC6137924; doi:10.1186/s40249-018-0473-9)
Supplement: Supplementary file 4 — List of included references. (DOCX 52 kb) [file 40249_2018_473_MOESM4_ESM.docx]

| **Authors, year** | **Country (region)** | **Surveillance objectives** | **Type of intervention** | **Overview of results (effectiveness/rapidity)** | **Lessons learned / recommendations** |
| --- | --- | --- | --- | --- | --- |
| Adams et al., 2016 [14] | Puerto Rico (USA) | To survey the spread of Zika virus | Protecting pregnant women, controlling the mosquito vector, and expanding access to the full range of voluntary contraceptive options for women and men | Adult mosquito populations in and around sprayed homes remained comparable to counts in and around unsprayed homes. During the outbreak, 16,522 patients were evaluated and 5,351 (32%) had laboratory evidence of current or recent Zika virus infection. Confirmed or presumptive Zika virus infection among symptomatic pregnant females increased from 8% to 41%, and persons with confirmed or presumptive Zika virus infection among symptomatic males and non-pregnant females increased from 14% to 64%. | Intensified vector control measures, including an integrated vector management strategy, are needed to help reduce disease spread. Clinicians who suspect Zika virus disease in patients who reside in or have recently returned from areas with ongoing Zika virus transmission should consider testing for Zika virus and report cases to public health officials. |
| Akoua-Koffi et al., 2002 [15] | Ivory Coast (Africa) | To detect cases for vaccination and to guide vector control | Case definition, diagnostic strategy (serology or biomolecular confirmation), larval index, imagos trapping | Weak for virus detection. Good for vaccination strategy. Positive in terms of implementation of a viral surveillance system. | Implementation of viral surveillance and establishment of a vaccination program for adults |
| Almeida et al., 2014 [16] | Brazil (Latin America) | To prevent transmission of yellow fever virus (YFV) to human populations | Vaccination of non-immune human populations living in the same area if they are not vaccinated | Surveillance in non-human primates was unable to predict the 2008–2009 outbreak or the rapid YFV transmission, although it detected YFV circulation in non-human primates in October 2008 before human cases occurred | Lack of preparedness or familiarity with such outbreaks in the region; better communication of YFV recommendations to travellers is also needed (they have to be vaccinated). |
| Alvarez Valdés et al., 2007 [17] | Cuba (Latin America) | To limit dengue transmission | Control of dengue based on community-based participation | An automated database with indicators and thematic map outputs made risk stratification for dengue and its vector possible. Additionally, 17 groups of neighbours were organized. | The comprehensive surveillance system for dengue developed in the project was an important tool for decision-making at the local level. |
| Arce et al., 2013 [18] | Spain (Europe) | To detect outbreaks of leishmaniasis and to set up the riposte | Questionnaire administered by telephone, sandfly trapping, surveillance of dogs by veterinarians, monitoring of other potential reservoirs, environmental sanitation | 35.9% visceral leishmaniasis and 64.1% cutaneous leishmaniasis; *Leishmania infantum* was identified as the agent, *Phlebotomus perniciosus* as the vector, hare as the secondary reservoir | A stable wild transmission cycle with hare linked to urban outskirts was identified, as well as extent of the vector distribution because of environmental changes. |
| Azil et al., 2015 [19] | Malaysia (Asia) and Australia  (Oceania) | To suppress vector population to prevent dengue fever | Vector control (traps) | Cairns: BG-Sentinel trap was a favoured method because of its user-friendliness, but not as cost-efficient as the sticky ovitrap. Kuala Lumpur: Mosquito Larvae Trapping Device was perceived as a solution for the inaccessibility of premises to larval surveys; Malaysia: larval survey method was retained for prompt detection of dengue vectors | To be successful, surveillance needs to include not only technical, quantitative evaluations of methods’ performance but also an appreciation of how amenable field workers are to using particular methods. |
| Bacon et al., 2008 [20] | USA | To detect Lyme disease early to enable early treatment | Prevention by: using insect repellents against ticks; daily self-examination for ticks; reduction of tick abundance around private homes and in recreational areas by removing brush and leaf litter; creating a buffer zone of wood chips or gravel between forests and lawn; applying acaricides; and excluding deer; use of single dose of doxycycline as prophylaxis in case of bite | Increase in annual cases of Lyme disease between 1992 and 2006, with a focus in north-eastern and north-central states, highlighted the need for improved surveillance; review of the case definition | Intensive surveillance methodologies, active population-based surveillance and use of non-human data (e.g., serologic testing of dogs and surveillance for vectors) are recommended for a better understanding of this emerging infectious disease. |
| Barbu et al., 2014 [21] | Peru (Latin America) | To prevent reinfestation of households by bugs to prevent. Chagas disease | Deltamethrin treatment of infested households | A single treatment was successful in 98.7% of infested households; a strong reduction in infestation attributable to the treatment and a strong effect of infestation on participation in the treatment were observed | The negative interaction between time since treatment and non-participation in the treatment phase suggests recolonization from non-participating households to their neighbours; identification of non-participating households appears as a priority to sustainable control |
| Blackmore et al., 2003 [22] | USA | To evaluate the spread of the West Nile virus (WNV) | Use of Sentinel Chicken Arboviral Surveillance Network | The 2001 WNV outbreak in Florida differed from the 1999−2000 outbreaks described in New York and surrounding areas; mortality in birds, particularly among crows and blue jays, was high; a number of Florida mosquito species were infected with WNV | Sentinel chicken surveillance will become a useful predictor of WNV activity; surveillance of arboviral encephalitis cases in horses will remain a valuable tool in assessing the risk for human WNV exposure; corvid mortality was the most sensitive predictor of WNV activity; the relative importance of mosquitoes as human virus vectors remains to be determined. |
| CDC, 2003 [23] | USA | To limit the transmission and the spread of West Nile Virus (WNV) | Vector control and sensitization of the population with information and policy actions | This was the first recognized occurrence of WNV in the Western hemisphere. It required the cooperation and coordination of many public and private partners, including government agencies. A panel of laws was promoted for vector control. *Culex* species played an important role in transmission in New York City and elsewhere in the USA. *Aedes japonicus* was detected recently, and research is needed to determine flight range and feeding behaviour and to better understand the risk for transmission to humans. | Counties where WNV transmission occurred in 1999 have to maintain surveillance and continue larval mosquito-control, such as controlling larval mosquito habitats, particularly around homes in suburban and urban areas, and monitoring of *Culex* larval habitats regularly for mosquito breeding. Public education and outreach programs to reduce mosquito breeding sites around the home have to be reinforced and use of personal protective measures encouraged. |
| Chaki et al., 2012 [24] | Tanzania (Africa) | To monitor malaria transmission | Vector control by larviciding programme | Different community-based systems of mosquito adults trapping were implemented | Community-based systems of surveillance can be improved by more sensitive traps but they already showed their efficacy |
| Chanda et al., 2012 [25] | Zambia (Africa) | To document significant reductions in malaria cases and deaths in all ages | Distribution of LLINs and IRS to households to reduce malaria transmission | Significant reduction with IRS in urban districts but not significant with LLINs in rural districts. A similar pattern was observed for case fatality rates. No substantial difference was detected in overall reduction of malaria cases between districts implementing IRS and LLINs | Using routine surveillance data to determine the temporal effects of malaria control is an important method for malaria monitoring and evaluation |
| Chang et al., 2009 [26] | Nicaragua (Latin America) | To monitor indices of larval infestation and density: home index, Breteau index, deposit index, and neighbourhood block index | Vector control based on source reduction according to visual imagery of the location of dengue cases and locations of potential larval development sites | A geo-referenced satellite base map was produced using satellite imagery freely available from Google Earth. Using aerial mapping of cases and likely larval development sites, high indices are colour-coded and statistically analyzed per neighbourhood block leading to greater spatial accuracy, in addition to precisely mapping larval development sites, homes of patients, and reference points. | Using a mapping system significantly increases the amount of information collected, ultimately saving time and money. Mapping of the area fumigated daily with gasoline and cypermethrin allows central administrators in Managua to recognize the extent of the interventions. The map can be used for other diseases (cholera, for example), or to identify deficiencies in health access, or improve water access |
| Cheung & Fok, 2009 [27] | China, Hong Kong (Asia) | Development of control actions against dengue fever | Detection of presence and distribution of mosquitoes by ovitraps | Reduction of the entomological indices. *Ae. albopictus* was widely distributed with high ovitrap indices, indicating the presence of persistent breeding grounds that needed continuous attention. *Ae. aegypti* was not detected in all the areas covered by the community and port surveillance programs. | Timely release of dengue vector surveillance results to all parties concerned through the GIS and to the public by press releases facilitated prompt remedial actions on vector control. Health education to sustain public participation in prevention and control of the vector continued to be one of the key elements in the mosquito prevention program |
| Chisha et al., 2015 [28] | Zambia (Africa) | To reinforce good case management and to ensure timely, reliable data are available to guide targeting of limited malaria prevention and control resources | Enhanced surveillance with data reporting, feedback, and laboratory quality assurance | 1) Better reported malaria data; 2) prospective data collection using improved forms; 3) improved quality assurance for diagnostic test results; and 4) consistent and timely reporting and feedback to the district and facility staff of results of prospectively collected information and quality assurance measures. | Clear benefits: a significant increase in the number of suspected cases being tested and a decrease in reported cases going unconfirmed. The enhanced surveillance program likely contributed to increased confidence in clinicians’ use of parasitological confirmations among patients. |
| Daudens et al., 2009 [29] | French Polynesia (Oceania) | To detect the occurrence of dengue outbreaks, to guide vector control actions and to identify circulated serotypes | Epidemiological evaluation of dengue fever surveillance in French Polynesia and vector control | Early detection of disease emergence through the sentinel network and monitoring of the outbreak thanks to hospital-based surveillance | Risk of re-emergence of a dengue genotype in the absence of circulation of another serotype for a few years. |
| Delisle et al., 2015 [30] | France (Europe) | To detect local transmission of chikungunya fever and to guide vector control actions | Enhanced passive surveillance (for case confirmation), active case finding, and entomological investigation | Cases were rapidly detected and confirmed. | Implement vector control as soon as several autochthonous cases are suspected. Implement vector control around imported cases in areas where the presence of the vector is known. |
| Díaz, 2012 [31] | Cuba (Latin America) | To reinforce dengue control and prevention | Vector control (source reduction) and detection of cases | Decrease in the number of unprotected water sources and in the proportion of unhygienic backyards; environmental improvements in public spaces; increase in number of febrile cases detected; reduction in the percentage of households that refused to allow inspections; increase in the community’s capacity to solve a problem on its own; increase in the active participation of agencies. | Success was due to (1) the training process implemented at the local level from the beginning of the project, which empowered the community and stakeholders in the use of the system tools; and (2) the collaborative process used by the health researchers, who gradually withdrew but stayed on as advisors to the management group. |
| EANMAT, 2001 [32] | Kenya, Uganda, Rwanda, Tanzania (Africa) | To adapt antimalarial treatments | Network of sentinels able to monitor effectiveness of malaria treatments | Treatment effectiveness data generated by EANMAT were used to evaluate and modify antimalarial treatment policies in all member states. | The EANMAT experience suggests that country response can be optimized by pooling resources and sharing experience and data through a small network (3–5 countries) |
| Espinoza et al., 2014 [33] | Bolivia (Latin America) | To prevent infestation of households by bugs to control Chagas disease | Insecticide spraying (mainly alpha-cypermethrin) | The program showed impressive effectiveness (immediate decrease) but residual infestation (no increase but no decrease after 2004); foci were widespread and will require long-term action. | Findings underscored the need for fully operational, long-term entomological-epidemiological surveillance systems based on public health policies capable of galvanizing sustained (and sustainable) preventive action. |
| Fine & Layton, 2001 [34] | USA | To improve the nation’s preparedness for a bioterrorist attack or any other large-scale infectious disease outbreak | Reduction of the mosquito adult population, mass distribution of mosquito repellant, massive public education | The public health response required rapid reduction of the adult mosquito population. Human, avian, and mosquito surveillance are necessary to evaluate the effectiveness of measures. The environment has to be cleaned to eliminate mosquito breeding sites. Education effort was conducted to provide information about the pesticides used, and the personal protective measures recommended against mosquitoes. Medical providers were also informed. All these efforts required intensive coordination and communication. | The experience with the WN virus outbreak offers practical lessons in outbreak detection, laboratory diagnosis, investigation, and response that might usefully influence planning for future infectious disease outbreaks |
| Flacio et al., 2015 [35] | Switzerland (Europe) | To control the spread of *Aedes* mosquitoes coming from Italy | Ovitraps supported by reports from the population; integrated control measures, including removal of breeding sites, larvicide applications with Bti or DFB and, in some circumstances, adulticide applications with permethrin | Findings showed new breeding sites for the mosquitoes that should be surveyed and controlled; active participation of the population in managing breeding sites, combined with regular treatments of public catch basins, were the key measures to control *Ae. albopictus*; a novel insecticide (Vectomax^®^) was also used. | Active participation of the population could be further improved by specific educational programs addressed to schools; authorities had time to deal with potential emergencies competently based on experience and up-to-date information. The *Ae. albopictus* surveillance in Ticino also served as the basis for development and implementation of the current extended surveillance at the national level in Switzerland. |
| Flamand et al., 2011 [36] | French Guiana (Latin America) | To specify the roles and missions of all stakeholders in integrated vector management, epidemiological surveillance, laboratory diagnosis, environmental management, clinical case management, and communication | Diagnosis of suspected cases of dengue fever coming from different medical sources | The validity of the surveillance system and its performance in monitoring dengue patterns in the whole country of French Guiana were validated, as well as its ability to detect outbreaks and to provide real-time information to health authorities. | Recent outbreaks showed that the implementation of the PSAGE at a region-wide level was not relevant considering the significant distances between municipalities. Future challenges and developments should focus on smaller territories by spreading the PSAGE across relevant spatial units. |
| Gavaudan et al., 2013 [37] | Italy (Europe) | To limit the spread of *Ae. albopictus* | Ovitraps and larvicide treatment with DFB or Bti (breeding site inextinguishable) and, if necessary, use of pyriproxyfen as adulticide | Climatic factors will not limit the spread of the local Pesaro *Ae. albopictus* population. Monitoring appears as a fundamental tool for effective mosquito control. The system, based on ovitraps, is economic and effective, and made it possible to measure the abundance and diffusion of the *Ae. albopictus* population. | To ensure the reliability of pest control systems, control and monitoring should be performed by different interested parties, including the population. Knowledge of vector control methods and their use must be disseminated among politicians and administrators. |
| Geissbühler et al., 2009 [38] | Tanzania (Africa) | To monitor malaria transmission | Use of microbial larvicides in addition of mosquito net coverage and treatment of cases | Larviciding reduced malaria infection risk among children ≤5 years of age and provided protection at least as good as personal use of an insecticide treated net | A well-implemented larval control can be highly efficacious as a component of an integrated vector management package which also includes ITNs in urban areas. The community-based larval control program applied Bti on a substantial operational scale(128,000 residents protected) to achieve a dramatic reduction of  malaria prevalence |
| Gitonga et al., 2010 [39] | Kenya (Africa) | Evaluation of malaria control | Diagnosis of cases, vector control and IEC | The overall prevalence of infection was 4.3% with a great variability among schools. Reported use of ITNs varied markedly across the country, and was <20% in the majority (55.0%) of schools. Disappointingly, only 11 schools reported ITN use >60%. Only a few schools reported having conducted any malaria control activities. | A cheaper and rapid complementary approach to household surveys would be to use the existing school system for school based malariometric surveys. |
| Gürtler, 2009 [40] | Argentina (Latin America) | To control Chagas disease | Bug detection and insecticide spraying; screening blood donors; diagnosis, etiologic treatment and medical care for chronic patients | The needs are to integrate vector and disease control in a single strategy. Sustained and continuous coordination between governments, agencies, control programs, academia and the affected communities is the basis of the success of the future strategy. | Sustainable suppression of bug infestation and *T. cruzi* transmission can be reached through integrated disease management. Vector control should be combined with active case detection and treatment to increase impact, cost-effectiveness, and public acceptance in resource-limited settings. |
| Handing et al., 2002 [41] | China (Asia) | To consolidate the elimination of schistosomiasis over the long term | Consolidate surveillance: survey and reporting; prevention; management and control; maintain alarms, remove dangers, modify environments; engineering;  scientific approach: antigen plate, quarantine, investigation, longitudinal surveillance | In the past 15 years, no single schistosomiasis case was found. Ten cases coming from other provinces were found to have histories of schistosomiasis; ten were parasitologically confirmed as schistosome infected subjects. They were all treated with praziquantel and the spread of the source of infection was prevented. Among the 2000 cattle examined, none was found infected. | Schistosomiasis can be eliminated from large areas and the elimination can be consolidated in the long term along with economic development and with intensive and unremitting control efforts. |
| Hapuarachchi et al., 2016 [42] | Singapore (Asia) | To control dengue transmission by understanding the determinants of the outbreaks and create an early warning system | 1) Enhanced case surveillance by using RDT. 2) Virus surveillance. 3) Entomological surveillance. 4) Understanding the relationship between environmental parameters and outbreak risk. | Epidemic resurgence of dengue fever in Singapore in 2013 was multi-factorial. A switch in the dominant serotype from DENV-2 to DENV-1 in March 2013 signalled a potential spike in cases, and the alert was corroborated by an increase in average *Aedes* house index. There was widespread distribution of *Ae. aegypti* in the country; 1/3 of the monitored sites remained at high risk of DENV transmission in 2013, | A multi-pronged approach backed by epidemiological, virological, and entomological understanding paved the way to moderating the case burden through an integrated vector management approach. |
| Hashimoto & Yoshioka, 2012 [43] | South and Central America (Latin America) | To control Chagas disease | Bug collections and insecticide treatments | Prevalence of Chagas disease has declined in Latin America. The affected population was reduced from 16–18 million in the 1980s to 8–9 million today. Along with remarkable progress in medical knowledge and practice, effective interventions have been implemented mainly to interrupt disease transmission. Much of the effort and resources were dedicated to controlling the vector, which was responsible for more than 80% of all transmission. | More stakeholders need to be involved at the local health facilities and community levels to control a larger number of infested houses; response to community bug reports needs to be improved in terms of cost, coverage, and time; monitoring needs to be done at a regional scale; the unit of certification can be divided into smaller scales, such as departmental and provincial levels, house improvement, environmental management. |
| Hernández-Ávila et al., 2013 [44] | Mexico (Latin America) | To identify at-risk areas for vector control against dengue | Web-based, geographically enabled, dengue integral surveillance system for collection, integration, analysis and reporting of geo-referenced epidemiologic, entomologic, and control interventions data | Timely information acquired by GIS and implementation of actions based on surveillance data (epidemiological data used for planning next season’s control operations and entomological surveillance data used to assess a posteriori anti-mosquito interventions). Better access to information related to dengue (surveillance and control) provided to different stakeholders. | Operatively, data input screens must be constructed to be as simple as possible and user-friendly. Adopting the system for routine use requires changes in perceptions about its usefulness and in the operative culture of its potential users |
| Huy et al., 2010 [45] | Cambodia (Asia) | To monitor different trends in dengue (circulating serotype, incidence, gravity) and to guide vector control | 1) Passive reporting of clinically diagnosed cases. 2) Virological surveillance at five hospitals. 3) Sentinel surveillance for children in sentinel hospitals (three public hospitals and three non-profit private hospitals in four provinces) | The alert system for detecting epidemics was efficient for the 2007 major outbreak. | To assess vector control interventions at the village level, as dengue transmission is highly localized. In the absence of systematic laboratory diagnosis of dengue, surveillance programs should exclude patients with undifferentiated febrile illnesses to increase specificity of diagnosis. |
| Kampen et al., 2015 [46] | Europe | Enhancement of active surveillance and of communication between government, citizens, and scientists | Mosquito reporting by population | Passive surveillance does not replace active systems, but incorporating observations of the interested public (“citizen science”) in data collection is a good complement with low cost and possible increase in people’s knowledge. It calls for cooperation across borders. The number of mosquito species known was increased. | Passive surveillance is appropriate to raise awareness and improve knowledge among citizens on entomological issues, invasive species, and associated public health problems. |
| Karema et al., 2012 [47] | Rwanda (Africa) | To monitor malaria transmission | Vector control and early detection of cases | A greater than 50% decline in malaria cases and deaths was observed following scale-up of mainly ITN and ACT in Rwanda since 2005. The decline occurred among both children under 5 and those 5 years and above, while hospital use increased and suitable conditions for malaria transmission persisted. Declines in malaria indicators in children under 5 were more striking than in the older age groups. The effect of community case management of malaria on trends in district hospital malaria cases appears to have been small. | The resurgence in cases associated with decreased ITN coverage in 2009 highlights the need for sustained high levels of anti-malarial interventions in Rwanda and other malaria endemic countries. |
| Kay & Nam, 2005 [48] | Vietnam (Asia) | To control dengue transmission | Prioritised control based on the larval productivity of major habitat types. Use of predacious copepods Mesocyclops as a biological control agent, delivered by community health workers (local leaders, health volunteer teachers, and schoolchildren) | In total, 386,544 people have been protected from dengue, since none of these communes has had cases since 2001. Similar results were observed in the south. | Preliminary assessment of community knowledge, attitudes, and practice made it possible to establish that dengue was viewed as a serious problem. |
| Kelly et al., 2013 [49] | Solomon Islands and Vanuatu (Oceania) | To identify malaria cases and organize the riposte | Case detection, early treatment | As a proof of success, SDSS-based geospatial surveillance-response systems currently remain in operation in all three elimination provinces. Different stages of progress in each of the elimination provinces were observed. So the SDSS-based approach to surveillance-response can use GIS to support the rapid mobilization of appropriate response interventions. | Community engagement and participatory surveillance have to be enhanced to encourage: early treatment seeking behaviour and community level vigilance; accurate diagnoses; timely and effective reporting of cases from the health facility to provincial level via suitable communication channels; and robust proactive case detection and increased vigilance in high-risk priority areas, such as known populations of high mobility (e.g. logging and mining camps) and common entry points (e.g. sea and air ports). Use of sensitive field-based molecular methods is required to effectively detect low-level infections. |
| Khosa et al., 2013 [50] | South Africa (Africa) | To decrease malaria transmission | Vector control and case treatment using artemisinin-based combination therapy (ACT) | An overall decreasing trend in malaria morbidity was observed during most seasons of the study period, mostly after a peak during the 2007–2008 season, which may be due to the synergistic effect of the scaling-up of IRS in the country and the introduction of ACT for the treatment of uncomplicated malaria in 2004. The decreasing trend is in line with patterns reported elsewhere in Limpopo Province and in other malarious provinces in South Africa. | The malaria control program in Limpopo should strengthen the surveillance, reporting, and capture of data in the provincial malaria information system. The actual population of household structures in Mutale municipality has to be well documented. South Africa should establish or strengthen cross-border malaria control collaborations beyond Mozambique and Swaziland, to minimize malaria importation. |
| Kuan & Chang, 2012 [51] | Taiwan (Asia) | To prevent some of the local transmission chain of dengue | Active detection of dengue importations in airports | Overall, 44.9% of the confirmed imported dengue cases with apparent symptoms were detected by the thermal screening program. Country origins could be known. Airport dengue screening also offers an opportunity to create a model for predicting the potential magnitude of all dengue importations. The non-endemic status of dengue in Taiwan was confirmed. A distinct impact of the dengue importations on community epidemics was observed due to ecological heterogeneity. | To curb potential dengue transmission, the authors strongly recommend reinforcing mosquito bite prevention among travellers or residents returning from dengue-endemic areas, or those living in dengue-competent or dengue-endemic hotspots. |
| La Ruche et al., 2010 [52] | France (Europe) | To detect local transmission of dengue and to guide vector control actions | Enhanced passive surveillance (for case confirmation), active case detection, and entomological investigation | Rapid case detection and confirmation. | Possibility of autochthonous dengue transmission in Europe. Enhanced surveillance allows the reporting and confirmation of suspected cases to be accelerated. The laboratory network surveillance system appears to be the most sensitive routine system in France. |
| Larsen et al., 2015 [53] | Zambia (Africa) | To improve the routine surveillance to better capture symptomatic and asymptomatic malaria cases, to improve access to healthcare, and to enhance public health intervention | (Re)active case detection in connection with confirmed malaria cases | In Lusaka, 3,955 individuals were tested and only 66 were found positive by rapid diagnostic tests. | Development of a formal, paid community health workforce in Zambia will need to be considered. Using a simpler drug regimen would improve treatment compliance, especially for individuals with asymptomatic malaria infections. |
| Lee & Fok, 2008 [54] | China (Asia) | To determine the prevalence and distribution of Aedes | Dengue vector surveillance and control (ovitraps) | According to the results of the dengue vector surveillance in 2007*, Ae. aegypti* was not detected and the activity of *Ae. albopictus* was, in general, under control. The Monthly Ovitrap Indices were mostly lower than the averages of the past few years, except in July, when a surge in the ovitrap indices was observed. | Active participation of the government, local organizations, and the public was the key to success in controlling the dengue vector. |
| Lee et al., 2010 [55] | Singapore (Asia) | To detect an emerging serotype of dengue | Laboratory-based dengue surveillance (PCR) with serotyping and phylogenetic analysis (sequencer) of DEN2 virus | Early detection of new serotypes of the dengue virus and early activation of vector control. Authors did not assess the effectiveness of the control measures, but considered them to be efficient, based on the situation in 2005 | Dengue virus serotypes are rapidly replaced. Continuous importation of dengue viruses was observed with establishment at various levels. |
| Lee et al., 2016 [56] | USA | To organize the riposte against Zika and after its surveillance | 1) Current testing and epidemiologic surveillance for Zika virus. 2) Surveillance to detect local transmission. 3) enhancement of mosquito control. | Preparedness for local transmission of Zika virus involves a robust emergency response infrastructure, targeted public health messaging, human and environmental surveillance strategies, and an integrated epidemiologic, clinical, and environmental response. A Zika Testing Call Center was established, and geospatial analysis was used to identify neighbourhoods at risk. | Pregnant women and persons with a Zika-like illness who have been in Zika virus-affected areas should be tested for Zika virus infection. Providers should offer up-to-date information on the risk for birth defects so that pregnant patients can make informed decisions about pregnancy options. |
| Lopez, 2002 [57] | USA | To limit the transmission and spread of West Nile virus (WNV) | Vector control | The activities contemplated the aggressive elimination of water accumulations conducive to mosquito breeding and extensive application of larvicide to standing bodies of water, including all catch basins in the city, of which there are at least 135000. The plans also provided for the possibility of spraying adulticides into the air to kill mosquitoes | Several laws were adopted to enforce the elimination of the mosquito nuisance that were based on collaboration between different agencies, information of the population, and pesticide regulation. |
| Lukacik et al., 2006 [58] | USA | To limit the transmission and spread of West Nile virus (WNV) | Mosquito and virus surveillance | The number of pools tested dropped substantially from 2000 to 2001, although the number of counties conducting surveillance doubled from 21 to 43 of 57 statewide. WNV nucleic acid was detected in 814 pools of 10 species: *Cx. pipiens, Cx. restuans, Cx. salinarius, Ae. vexans, Oc. japonicus, Oc. sollicitans, Oc. trivittatus, An. punctipennis, Cq. perturbans,* and *Cs. melanura.* | The efficacy of the WNV surveillance system can be improved by entering mosquito collection data onto the HIN in a timelier manner, collecting and testing mosquito pools from highly populated areas of NYS, and focusing on gravid trap-based programs. Other improvements can be made by maintaining an infrastructure conducive to collecting, handling, and testing large numbers of mosquito pools, and testing for medically relevant arboviruses endemic to NYS, including WNV, Eastern equine encephalitis virus, Lacrosse encephalitis virus, and Jamestown Canyon encephalitis virus |
| Maciel-de-Freitas et al., 2014 [59] | Brazil (Latin America) | To provide an early warning system | Vector control based on community participation (source reduction, insecticides) | 56,837 homes, corresponding to 74% of all habitations, were visited in Boa Vista. 94,325 containers were removed or treated in the 22 monitored districts, leading to a slight reduction of mosquito infestation levels. The intensification of vector control measures resulted in a dramatic increase in the resistance status of mosquitoes to deltamethrin but no increase in resistance to temephos was observed. | The rapid increase of insecticide resistance alleles in *Ae. aegypti* field populations highlights the need to develop alternative strategies such as insecticide rotations and mixtures to delay the evolution of resistance. |
| Maia-Elkhoury et al., 2008 [60] | Brazil (Latin America) | To assess the prevalence of leishmaniasis in the whole country | Laboratory diagnosis of infection and disease in humans and dogs, treatment of patients, evaluation of the effectiveness of control strategies, and development of new technologies that could contribute to the surveillance and control of visceral leishmaniasis in the country | The mean incidence of visceral leishmaniasis in the past 12 years was 2 cases/100,000 inhabitants, and the case-fatality rate was 5.5%, representing an increase of 117% in 2005 (6.9%) when compared to the case-fatality rate in 1994. | Implementation of new control measures is needed (adequate treatment of human cases, monitoring and euthanasia of sero-reactive dogs, environmental management, and chemical control). |
| Moreno et al., 2011 [61] | Mexico (Latin America) | To limit dengue transmission | Field teams, mobilization of different actors (university, public authorities, local communities) | Dengue cases were reduced by more than 80% in 2010 as compared to 2008, with a better control of insecticides used. | The best way to prevent dengue is to control the vector; future success depends on vaccine development and genetic modification of the vector. |
| Mostashari et al., 2003 [62] | USA | To provide a spatial-temporal surveillance system in real time of West Nile virus (WNV) | Collection of data on dead birds and mosquitoes | Spatial-temporal cluster analysis of dead bird reporting data made it possible to initiate early larval control activities, prioritize birds for testing, and triage scarce mosquito-collection and laboratory resources. All these activities enabled more effective and efficient prevention of human disease caused by WNV. | The system may prove useful for early detection of other infectious disease outbreaks and for bioterrorism surveillance by using pre-diagnostic clinical or consumer data (“syndromic surveillance”). |
| Msellemu et al., 2016 [63] | Tanzania (Africa) | To monitor malaria transmission | Vector control (microbial larvicides, environment management (drain cleaning, mosquito-proof housing), high coverage of ITN) and treatment of cases using ACT | After a decrease in malaria transmission observed in 2008, recent studies showed an increase explained by different hypotheses: behavioural resistance of the vectors (LLIN), immunity fall in human population (because of chronic and no asymptomatic infections), private contractor for the LA, | The most immediate opportunity for progress towards malaria elimination is further scale-up of LA and mosquito-proofed housing. However, accelerated elimination of malaria from this urban setting will also probably require active, population-wide mass screen-and-treat or mass drug administration campaigns to cure chronic human infections. Evaluated trap designs tested during the program can be adapted for the surveillance of a variety of mosquito borne diseases including malaria, lymphatic filariasis and dengue fever |
| Naranjo et al., 2014 [64] | Ecuador (Latin America) | To be able to intervene in case of emergence of a vector-borne disease | Rapid detection of diseases and household visits with vector control (SNEM and AMCD) | Strengths: research-based operations and community participation; weaknesses: epidemiological-entomological surveillance and insecticide resistance; opportunities: capacity building, research collaborations and improved municipal services; threats: political, geographical situation, environmental and social constraints. | SNEM needs to improve its entomological component and AMCD needs to improve its epidemiological component; both organizations need to increase their financial capacity; integrated approaches have to be ensured; new technologies such as GIS may be added to improve risk assessment and disease control; stakeholders should strengthen investments in community education; collaboration with decision-makers (policy) is needed; MCP must be integrated into public health surveillance systems. |
| Nash et al., 2001 [65] | USA | To limit the transmission and spread of West Nile virus (WNV) | Active surveillance was implemented to identify patients hospitalized with viral encephalitis and meningitis | 59 patients were hospitalized with West Nile virus infection in the New York City area during August and September of 1999. The overall attack rate of clinical West Nile virus infection was at least 6.5 cases per million population, and it increased sharply with age. Most of the patients (63 percent) had clinical signs of encephalitis; seven patients died (12 percent). Muscle weakness was documented in 27 percent of the patients and flaccid paralysis in 10 percent. | Given the subsequent rapid spread of the virus, physicians along the eastern seaboard of the United States should consider West Nile virus infection in the differential diagnosis of encephalitis and viral meningitis during the summer months, especially in older patients and in those with muscle weakness |
| Nogareda et al., 2013 [66] | Solomon Islands (Oceania) | To control the outbreak | Clinical training seminars, vector control activities, implementation of diagnostic and case management protocols and a public communications campaign | 5,254 cases were diagnosed. DENV-3 was identified as the pathogen. *Ae. aegypti* and *Ae. Albopictus* were the vectors. | Continued nationwide enhanced surveillance and response activities are recommended, with particular attention needed at the provincial level; a national clean-up day should be declared by government. |
| Novello et al., 2000 [67] | USA | To limit the transmission and the spread of West Nile Virus | Extensive mosquito-control and risk-reduction campaign was initiated, including aerial and ground applications of mosquito adulticides throughout the affected areas | CDC issued guidelines to direct national surveillance, prevention, and control efforts (*2*) and provided funds to support these efforts in 19 state and local health departments where WNV transmission had occurred or where transmission would probably occur based on known bird migration patterns | Counties where WNV transmission occurred in 1999, but has not been identified in 2000, should maintain active surveillance for WNV and continue larval mosquito-control, such as controlling larval mosquito habitats, particularly around homes in suburban and urban areas and monitoring *Culex* larval habitats regularly for mosquito breeding |
| Palaniyandi, 2014 [68] | India (Asia) | To prevent dengue outbreaks | Source reduction of vector breeding habitats and fogging in the breeding areas | GIS makes it possible to update and map dengue prevalence in real time. It is a tool for dengue surveillance and public health information management, as well as a decision-making tool for controlling dengue epidemics at least 7 days in advance. | The system has to be based on public actors, researchers, and NGOs. The population needs to be informed. House visiting should be implemented through the town leaders. Data need to be entered into the GIS platform as soon as possible. |
| Pepin et al., 2013 [69] | Brazil (Latin America) | To directly measure adult female mosquito abundance in real time | MosquitoTRAP (and reporting data in Web-database) | Preliminary results from three cities (Tres Lagoas in Mato Grosso do Sul State, and Presidente Epitacio and Bastos in Sao Paulo State) during one season of MID implementation showed that this system is effective in decreasing dengue cases. Implementation of MID has reduced the incidence of dengue by 68%. However, the authors state that this result, higher than that obtained in Cambodia, is due to geographical differences and to the scale of implementation of MID, which was greater. | The cost-effectiveness of MID was higher in cities with annual incidences of more than 72 cases per year. Generally, MID is more effective than other strategies in reducing dengue cases, in that it saved about $7 million that would have been lost in wages. |
| Pepin et al., 2015 [70] | Brazil (Latin America) | To monitor weekly prevalence of gravid *Ae. aegypti* and *Ae. albopictus* city-wide in real time | Mosquito control strategy (larvicide and source reduction activities) | Neighbourhood-level averages of household vector density were a poor predictor of dengue-fever cases in the absence of accounting for interactions with human cases | For spatial prioritization of vector controls, city-wide spatial effects should be given more weight than within-neighbourhood or nearest-neighbourhood connections, to minimize city-wide cases of dengue fever. Future research should also include city-wide MID-virus data (infection of vector). |
| Pile, 2001 [71] | USA | To limit the spread of West Nile virus (WNV) | Elimination of breeding sites from individual yards | 1) Birds are the primary “amplifying” hosts of WNV. 2) Common symptoms include myalgia, headache, fatigue, and arthralgia. (3) Testing serum samples in both the acute and the convalescent stages is important to confirm WNV disease; (4) The use of repellents is needed | Use of birds as sentinel for viral activity; Monitoring virus activity in mosquito pools; Passive surveillance in both humans and horses showing signs of illness; Enhancement of mosquito control, particularly at the larval stage; Improvement of public education at the local level (elimination of breeding sites and use of repellents) |
| Quinde-Calderon et al., 2016 [72] | Ecuador (Latin America) | To evaluate the transmission of Chagas disease | Vector control (insecticide) by house visiting | Three distinct Chagas disease transmission scenarios were observed in Ecuador, with possible epidemiological changes of the disease transmission in the Amazon region, potentially associated with increasing deforestation; confirmation of the role of *T. dimidiata* and *R. ecuadoriensis* as primary vectors in the country | Need for reinforced epidemiological surveillance, sustained high vector control coverage activities to ensure continued success, novel vector control interventions and strong political commitment. |
| Raju, 2003 [73] | Fiji Island (Oceania) | To determine the entomological indices for controlling dengue | Source reduction, mediatic campaigns, training | Regular visits of health inspectors and health awareness created considerable awareness among populations to be more cautious about avoiding oviposition by mosquitoes. Community participation reduced the observed wet and dry containers. The numbers of drums breeding *Ae. aegypti* were reduced after community consultation and intervention messages. | Dengue control programs must include surveillance of mosquito populations to assess the effectiveness of source reduction campaigns and targeted mosquito control measures. The success of any prevention program depends on either convincing individuals to change their behaviour or changing the environment to remove factors that place individuals at risk of disease. |
| Raman et al., 2011 [74] | Mozambique (Africa) | To adjust national directives for malaria treatment | Evaluation of antimalarial treatment to allow an appropriate decision-making in case of resistance of the parasite | Results from this study support the decision taken to replace artesunate plus SP with artemether-lumefantrine, given the quintuple mutation nearing fixation. The high prevalence of this mutation also calls into question the useful therapeutic life of SP monotherapy for IPT in this region. | Results suggest the re-evaluation of IPT using SP in Mozambique and strongly support continued routine surveillance for antimalarial drug resistance markers to ensure the recent gains made by the malaria control program in Gaza Province are sustained. |
| Regis et al., 2013 [75] | Brazil (Latin America) | To monitor the *Aedes* population | Mass elimination of *Aedes* eggs and education for source reduction, coupled with GIS technology (pilot trial) | After two years, integrated control actions targeting mosquito eggs and larvae with social participation impacted the mosquito population and caused an overall reduction of around 77% and 90% in egg densities as compared to the pre-intervention period. Constructed from plastic bottles, the C-ovitraps embodied a symbolic value in addition to their didactic role and mobilized the community in all stages of the process. Adult aspirations contributed to the whole reduction and to improving the health of the population (reduction in the number of dengue infection cases). | The intervention’s success appears to depend on greater participation by the population. |
| Ritchie et al., 2002 [76] | Australia (Oceania) | To improve the rapidity of the response to reduce the number of dengue cases | Case recognition and vector control based on voice-response telephone system and internet, mosquito traps | Since the implementation of the DFMP 2000, five outbreaks of dengue that occurred in north Queensland have all been relatively easily contained, and very little locally-acquired dengue has been detected beyond the initial focus of activity. The number of cases acquired after the initiation of control efforts has been limited and the duration of outbreaks reduced. | The speed of response was increased. |
| Rocha et al., 2009 [77] | Peru (Latin America) | To identify dengue cases | 1) School-based surveillance: blood sample, plaque reduction neutralization assay, daily medical exams and tourniquet tests - IgM and ELISA. 2) Community-based surveillance with door-to-door visits | Community-based surveillance provided a viable alternative to school-based surveillance, capturing twice as many fever and symptomatic dengue infections relative to study population size than the school-based system while monitoring five times as many people using the same number of personnel and the same amount of resources. Several factors, including the research objective, site-specific dengue epidemiology, and cultural characteristics of the study population, will help determine the type of active surveillance system to implement | Site-specific dengue epidemiology, and cultural characteristics of the study population will determine the choice of the active surveillance strategy implemented. Based on results, community-based surveillance should be considered a viable methodology. |
| Santana et al., 2011 [78] | Brazil (Latin America) | To map the distribution of bugs and the risk of Chagas disease | Bug trapping by population and reporting | Entomological surveys carried out provided an estimate of the geographic triatomine distribution in the municipality of Salvador; half of the collected vectors carried *T. cruzi*, underscoring the risk for a Chagas epidemic | Attention should be drawn to the possible role of *P. geniculatus, T. tibiamaculata, Rhodnius prolixus, R. domesticus* and *T. vitticeps*, once these species start moving into human premises. The common presence of infected triatomes in urban areas underscores the increasing risk of a Chagas epidemic. The findings highlighted the need to develop guiding strategies for surveillance and control. |
| Seidahmed et al., 2012 [79] | Sudan (Africa) | To calculate density parameters of *Aedes aegypt*i in relation to the human population density | Sentinel sites, training, community mobilization (house inspection, health education campaign with pamphlets, radio message, written messages and video clips, lecture in social and sports location); routine program of inspection; space spraying, larviciding | 3,048 houses were inspected, resulting in the collection of 19,794 larvae and 3,240 pupae of *Ae. aegypti*. A total of 11,524 indoor water storage containers were examined; 2,536 were found to be infested with larvae and/or pupae of *Ae. aegypti*. Among all storage water containers, the key vessels were clay pots (75%) and plastic barrels (15%). | This work clearly shows the impact on dengue disease transmission of effective entomological surveillance with coordinated control intervention. Community mobilization was an integral part of the response plan. One of the challenges to community mobilization is compliance of household members to regularly scrub and filter their drinking containers. In a systematic review of dengue control programs, results showed that chemical interventions are ineffective, while educational campaigns seem to be effective. Moreover, the authors think chemical control should be a part of response plans in the case of dengue epidemics. |
| Sharma et al., 2014 [80] | India (Asia) | To control malaria by reducing vector population in urban areas | Use of larvicides, adulticides, larvivorous fishes, and treatment of infections | Increase of malaria may be related to: increasing and unplanned urbanization; poor disease surveillance activities in towns; intermittent water supply leading to bad storage behaviours; absence of health impact assessment leading to *Plasmodium falciparum* resistance; and inadequate health infrastructure | A new strategy is proposed based on: (i) detection and management of malaria cases and other VBDs; (ii) integrated vector management; (iii) capacity building and behaviour change communication; (iv) intersectoral coordination; and (v) legislative measures, building by-laws |
| Sharp et al., 2014 [81] | Puerto Rico (USA) | To detect chikungunya transmission | Definition of the medical procedure for chikungunya cases with respect to the procedure for dengue | 10,201 suspected cases (282 per 100,000 residents) were reported between May and August 2014. The identification of chikungunya patients through household investigations suggested the incidence of chikungunya was higher than demonstrated by passive surveillance data. | Information campaigns are required to increase knowledge of the disease among health care professionals and populations; PRDH and CDC recommended that health care providers manage suspected chikungunya cases as they do dengue because of the similarities in symptoms and increased risk for complications in dengue patients who are not appropriately managed. |
| Simarro et al., 2006 [82] | Equatorial Guinea (Africa) | To control the sleeping disease transmission | Detection and treatment of sleeping sickness cases | Given that no cases were identified since 1995 despite permanent passive case-finding and periodic active case-finding surveys (1997, 1999, 2001 and 2004), it was concluded that the Luba focus was no longer active. | With a clear community demand, strong political support, and availability of funds, the chance of success is very high. |
| Soriano-Arandes et al., 2014 [83] | Spain (Europe) | To follow all pregnant women originating from Latin America | Active surveillance and health promotion activities related to Chagas disease (interview, group workshop/sessions, community awareness campaign, audiovisual material) | 43 cases identified. The paediatrician recovered follow-up information from 31/35 identified newborns; the remaining 4 cases probably left Catalonia between birth and surveillance. Of the 31 recovered infants, 12 were regularly controlled at 9 months of age in hospitals outside the city of Hospitalet de Llobregat, but this information was not communicated to ASPCAT. Only 5/19 were correctly followed up by family paediatricians; the remaining 14 (73.7%) were therefore not correctly tested for *T. cruzi* at 9 months of age. | Screening for Chagas disease (CD) which is not transmitted by vectors in Spain but represents a public health problem in the country, in pregnant women or newborns provides an opportunity to detect further cases among siblings and relatives, as infections often occur in clusters. The poor control of newborns from CD-positive mothers in paediatric primary health care centres highlights the need for training in congenital CD addressed to those paediatricians. Experience to date suggests that the most effective strategies are those that promote face-to-face actions among influential groups organized into community action networks. |
| Succo et al., 2016 [84] | France (Europe) | To detect local transmission and to guide vector control actions | Enhanced passive surveillance (for case confirmation), active case detection, and entomological investigation | Rapid case detection and confirmation. | Adjustment of the perimeter for vector control and assessment of vector control effectiveness. |
| Suter et al., 2016 [85] | Switzerland and Italy (Europe) | To limit the spread of the vector | Ovitraps with Bti | In the urban environment, the *Ae. albopictus* egg density was 2.26 times higher in the non-intervention area, on the Italian side of the border, as compared to the intervention area in Ticino; the ratio in egg densities between the urban and sylvatic environment was twice as high in the non-intervention area. | The currently implemented surveillance and control program in Ticino has a positive impact. Public awareness is presumed to be a major component in reducing *Ae. albopictus* densities. |
| Taleo et al., 2000 [86] | Vanuatu (Oceania) | To identify dengue cases and implement a rapid response | 1) Surveillance: larval survey, training health care providers, reporting by phone. 2) Routine preventive measures: collect data on tires, clean-ups, larviciding, routine larval surveys. 3) Community-based control: education (media with TV, radio, posters), distribution and collection campaign for plastics bags, training of volunteers, meetings | During epidemics, control measures emphasized clinical case-management, health education, and mosquito control (larval breeding source reduction, larviciding and indoor focal house spraying in the homes of cases). During non-transmission periods, an active mosquito larval source reduction program with community participation was emphasized, along with training for health care providers and health education of the public. | Cooperation of the community is essential for outbreak prevention through larval source reduction programs. The Manples project has attracted the interest of other communities in Vanuatu and is being expanded to the main suburban communities. |
| Teng, 2001 [87] | Singapore (Asia) | To reduce outbreaks by identifying transmission hotspots or breeding sites | *Aedes* mosquito control (ovitraps) | New initiatives and tools exist to improve *Aedes* control: regular mosquito surveillance of housing estates and crowded places, redesigning of structural habitats, incorporation of heating elements in roof gutters, and tracking of behavioural changes. Health education must also be provided. | High-level support, inter-agency cooperation and understanding are the keys to the success of the dengue control program; vector control is crucial. |
| Terranela et al., 2006 [88] | Nigeria (Africa) | To demonstrate the transmission of Lymphatic Filariasis (LF) in the town and to set up mass treatment | Detection of LF cases and treatment | LF urban transmission may have occurred. | Neighbourhoods identified as at risk of LF were recommended to the federal Ministry of Health as sentinel sites for monitoring and evaluation of the city-wide MDA-based LF control program launched in Jos in late 2003. |
| Tissera et al., 2016 [89] | Sri Lanka (Asia) | To better control dengue illness | Reorganization of the control of dengue (enhanced diagnosis, improvement of clinical case management, and vector control with environmental measures) | Enhanced real-time surveillance for early warning allows ample preparedness for an outbreak. National guidelines with enhanced diagnostics have significantly improved clinical management of dengue, reducing the case-fatality rate to 0.2%. Proactive integrated vector management, with multisectoral partnerships, has created a positive vector-control environment. | Making the maximum effort to minimize outbreaks through sustainable vector control in the three dimensions of risk mapping, innovation, and risk modification will help to reduce morbidity. |
| Van Cauteren et al., 2012 [90] | France (Europe) | To limit the spread of chikungunya fever | Mandatory declaration and vector control around the cases | The surveillance systems did not register the same data. In 2011 imported cases were registered but no local transmission was observed; the metropolitan situation is mostly influenced by the epidemiological situation in the tropics, in particular in the French overseas territories. | The preparedness and response plan against the dissemination of chikungunya and dengue in metropolitan France should be adapted, due to the ongoing spread of *Ae. albopictus* towards other regions in metropolitan France, taking into account the available resources and the surveillance systems performance. |
| Xi-min Hu et al., 2008 [91] | China, Hong Kong (Asia) | To eliminate lymphatic filariasis | Mass treatment of the human population and vector source reduction | In cross-sectional and entomological surveillances, the last microfilaremia case was found in 1999, and no mosquitoes were found to be infected with filarial larvae. A total of 132 microfilaremia cases found in the longitudinal surveillance became negative within 10 years. Since 1997 no mosquitoes have been found to be infected with filarial larvae in the longitudinal surveillance. Serological surveillance of the population showed the mean positive rate of IFAT dropped from 10.63% in 1990 to 0.28% in 2000, which is similar to that of non-endemic areas. | Residual sources of infection after basic elimination of filariasis tended to be naturally eliminated and transmission of filariasis has been interrupted in Hainan Province. Filariasis transmission tended to be interrupted even without control measures when the microfilarial rate was below 1% and the microfilarial density less than 10 per 60 µl of blood. |
| Yoshikawa, 2013 [92] | Singapore (Asia) | To prevent arboviral diseases | Source reduction and insecticide by surveillance of residential areas, construction sites, and foreign workers’ quarters | The vector control program led to moderate disease prevalence and/or rapid control of domestic outbreaks | An effective vector control program requires control of urban development, a legal framework, public education, cooperation of residents, social responsibility of businesses, multicultural/multilingual communication with residents and foreign workers. |
